# Supplementary material for: Midbrain glutamatergic circuit mechanism of resilience to socially transferred allodynia in male mice
Source: Nat Commun. 2024 Jun 10;15:4947. doi: 10.1038/s41467-024-49340-8 (PMC11164890; doi:10.1038/s41467-024-49340-8)
Supplement: Supplementary file 1 — Supplementary Information [file 41467_2024_49340_MOESM1_ESM.pdf]

Midbrain glutamatergic circuit mechanism of resilience

to socially transferred allodynia in male mice

SUPPLEMENTARY FIGURES AND TABLES

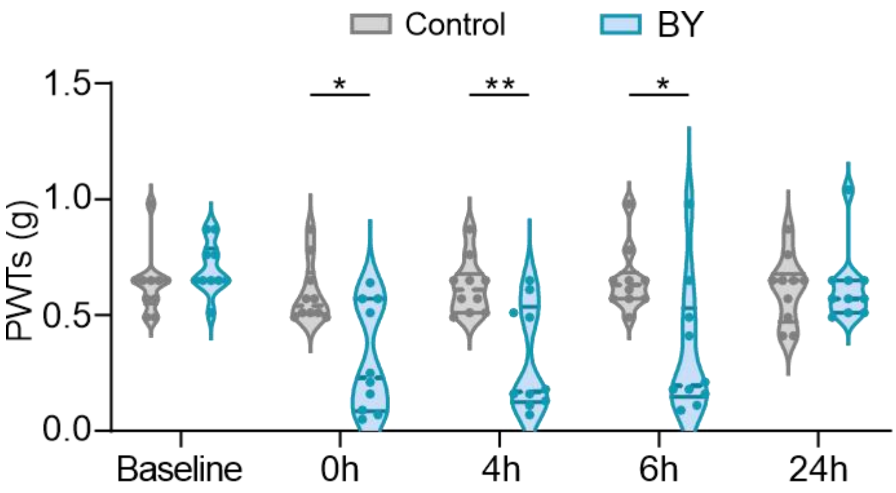

Supplementary Fig. 1 | Time course and quantitative data of von Frey tests for PWTs at different time points before and after 1-hour social contact in control and bystander mice (Control, n = 10 mice; BY, n = 10 mice). Control versus BY, $P_{BL} = 0.7615$, $P_{0h} = 0.0243$, $P_{4h} = 0.0089$, $P_{6h} = 0.0481$, $P_{24h} > 0.9999$. BY, bystander mice. The data are presented as the mean ± s.e.m. $^{*}P < 0.05$, $^{**}P < 0.01$. Data analyzed by Two-way ANOVA with Sidak's multiple comparisons test. Statistical details are presented in Supplementary Table 5. Source data are provided as a Source Data file.

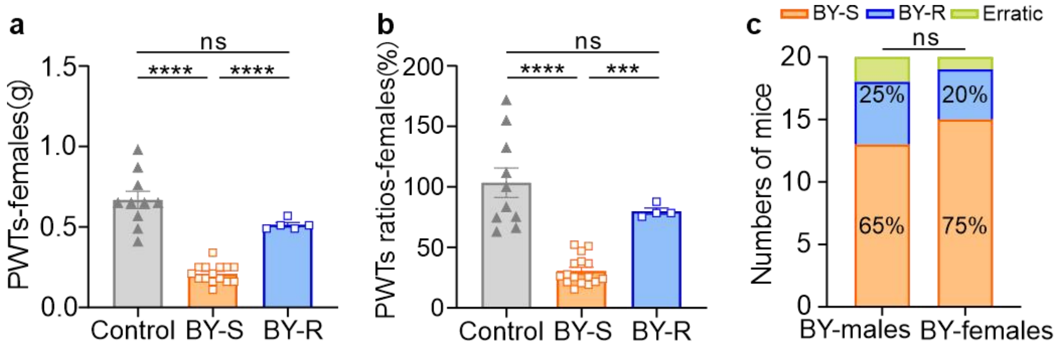

Supplementary Fig. 2 | Segregation of susceptible and resilient subpopulations in female BY mice. a, PWTs of female mice (*n* = 10, 15 and 5 mice). Control versus BY-S, $P = 2.9 \times 10^{-11}$; Control versus BY-R, *P* = 0.0530; BY-S versus BY-R, $P = 6.8 \times 10^{-7}$. **b,** PWTs ratios of female mice (*n* = 10, 15 and 4 mice). Control versus BY-S, $P = 1.2 \times 10^{-7}$; Control versus BY-R, *P* = 0.3917; BY-S versus BY-R, *P* = 0.0004. **c,** Percentages of BY-S and BY-R subpopulations in both male (*n* = 13, 5 and 2 mice) and female mice (*n* = 15, 4 and 1 mice). *P* = 0.7483. The data are presented as the mean ± s.e.m. ****P* < 0.001, *****P* < 0.0001, ns: no significance. Data analyzed by (**a, b**) one-way ANOVA with Tukey's multiple comparisons test; or (**c**) chi-square test. Statistical details are presented in Supplementary Table 5. Source data are provided as a Source Data file.

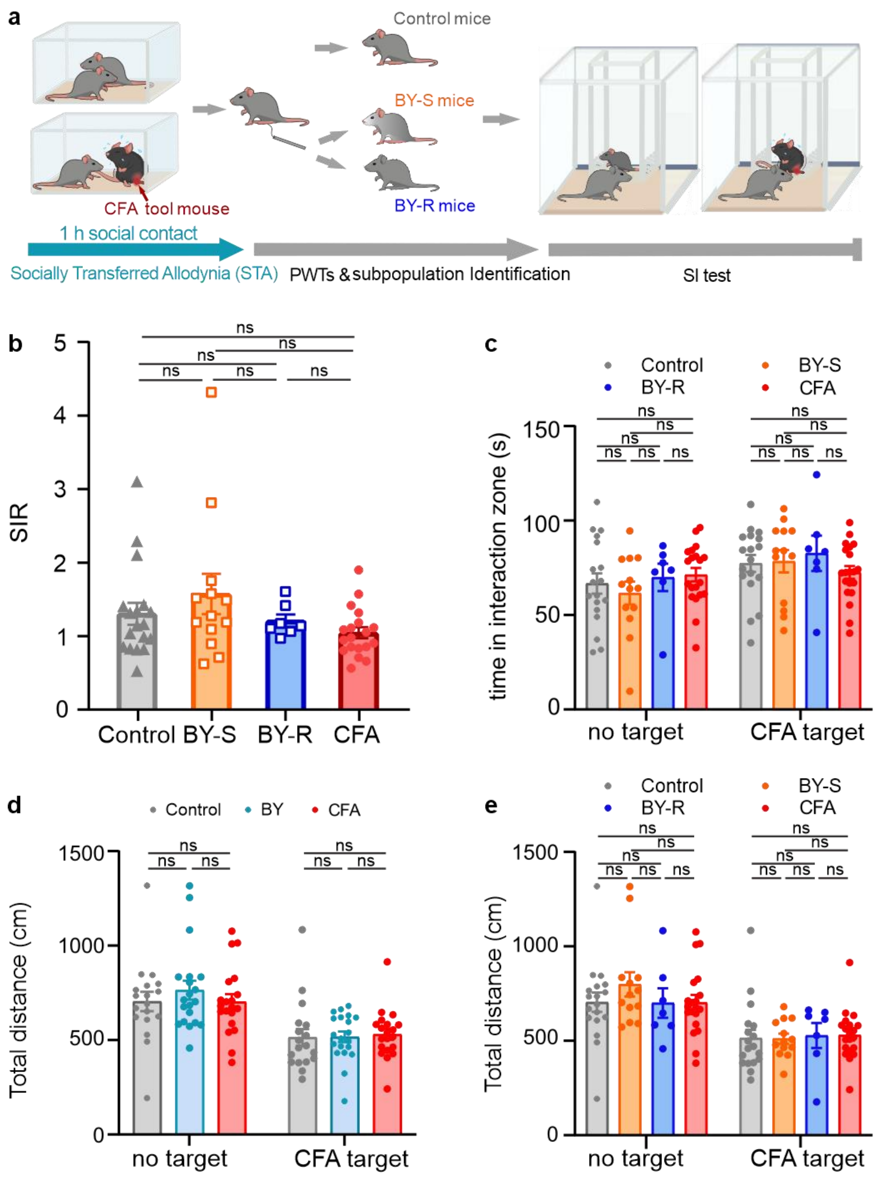

Supplementary Fig. 3 | BY-S and By-R mice displayed similar social interaction with CFA mice. a, Experimental timeline. **b,** Social interaction ratios (*n* = 18, 13, 7 and 20 mice). **c,** Interaction zone time (*n* = 18, 13, 7 and 20 mice). **d,** Total distance traveled (*n* = 18, 20 and 20 mice). **e,** Total distance traveled (*n* = 18, 13, 7 and 20 mice). SI, social interaction. The data are presented as the mean ± s.e.m. Ns: no significance. Data analyzed by (**b**) Kruskal-Wallis test with Dunn's multiple comparisons test; or (**c, d, and e**) two-way RM ANOVA with Tukey's multiple comparisons test. Statistical details are presented in Supplementary Table 5. Source data are provided as a Source Data file.

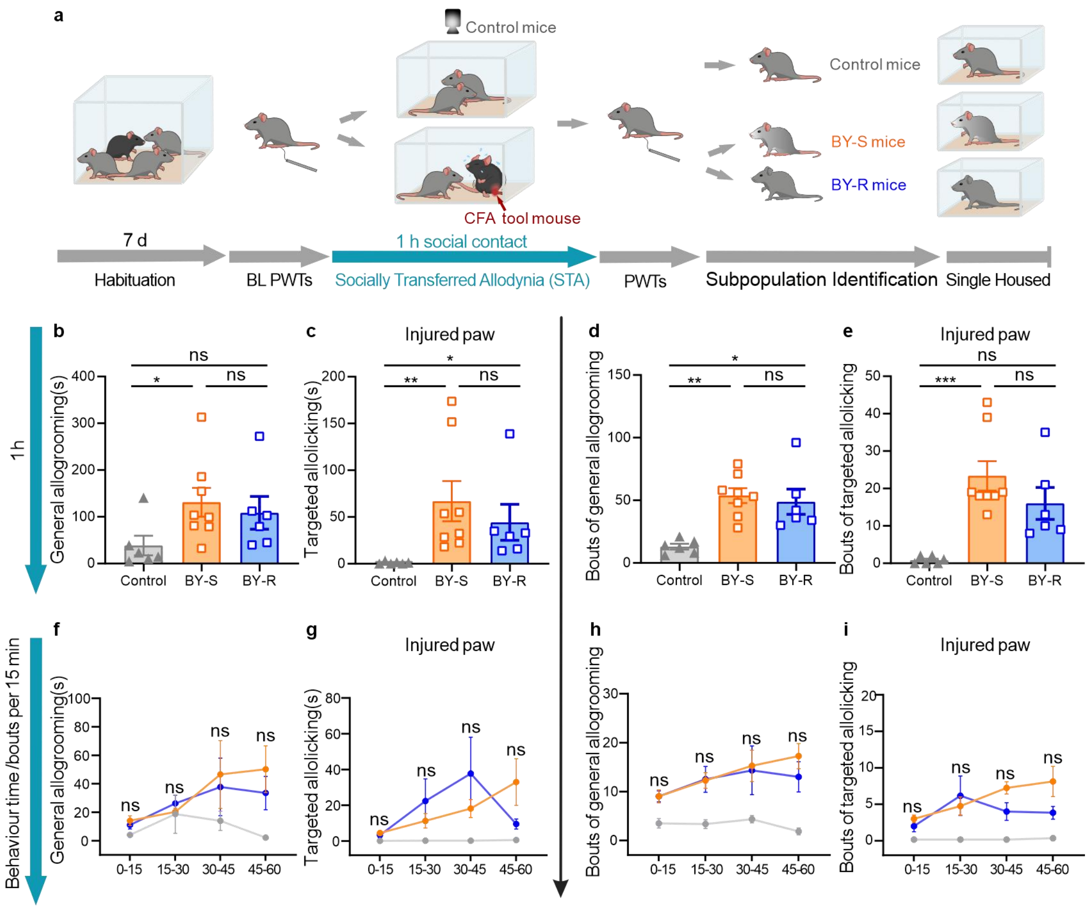

Supplementary Fig. 4 | General allogrooming and targeted allolicking during the STA paradigm. a, Experimental timeline. **b-e,** Total duration (**b, c**) and bouts number (**d, e**) of allogrooming and targeted allolicking behaviors during 1-hour STA (*n* = 6, 8 and 6 mice). (**b**) Control versus BY-S, *P* = 0.0397; (**c**) Control versus BY-S, *P* = 0.0020; Control versus BY-R, *P* = 0.0288; (**d**) Control versus BY-S, *P* = 0.0020; Control versus BY-R, *P* = 0.0291; (**e**) Control versus BY-S, *P* = 0.0010. **f-i,** Cumulative duration (**f, g**) and bout number (**h, i**) of allogrooming and targeted allolicking behaviors in BY-S, BY-R, and control mice measured every 15-minute (*n* = 6, 8 and 6 mice). The data are expressed as the mean ± s.e.m. **P* < 0.05,***P* < 0.01, ****P* < 0.001, ns: no significance. Data analyzed by (**b-e**) Kruskal-Wallis test with Dunn's multiple comparisons test; or (**f-i**) Two-way RM ANOVA with Tukey's post hoc test. Statistical details are presented in Supplementary Table 5. Source data are provided as a Source Data file.

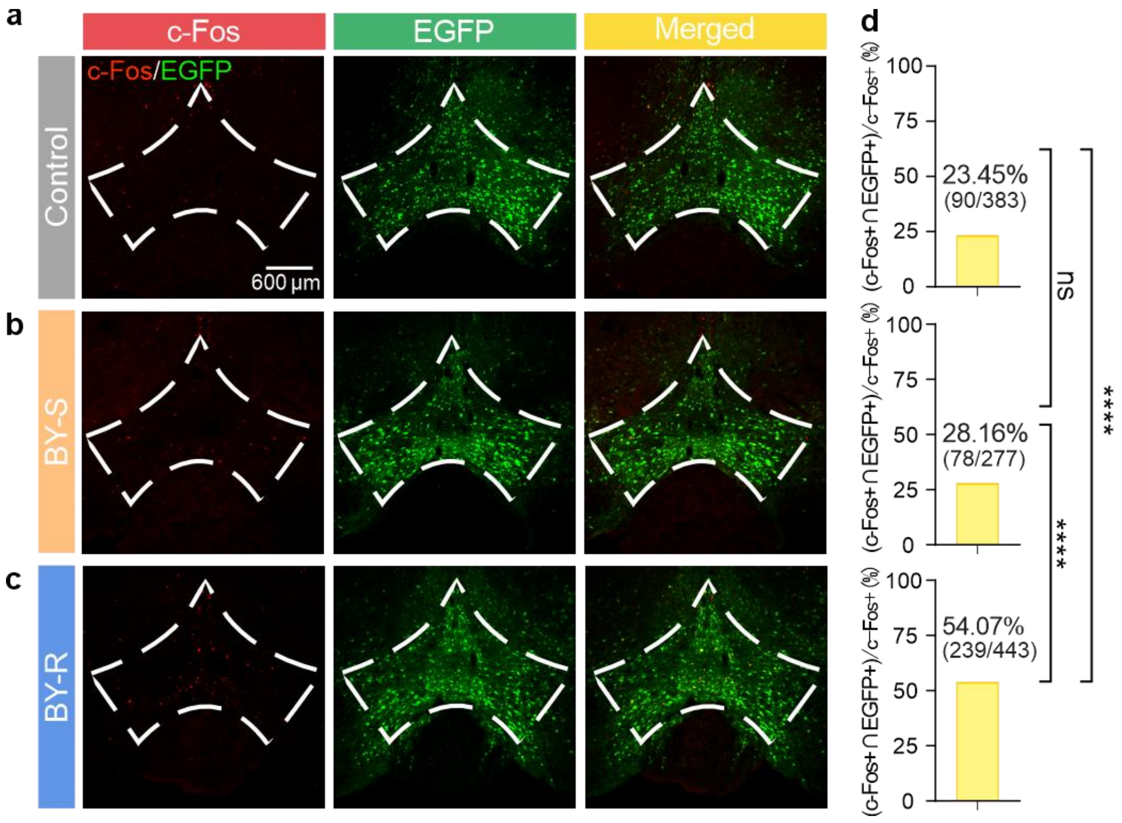

Supplementary Fig. 5 | Specific activation of VTA glutamatergic neurons in the resilient mice. a, Representative immunofluorescent images and the proportion of c-Fos protein expression in VTA glutamatergic neurons from control mice (n = 9 slices from 3 mice). **b,** Representative immunofluorescent images and the proportion of c-Fos protein expression in VTA glutamatergic neurons from BY-S mice (n = 8 slices from 3 mice). **c,** Representative immunofluorescent images and the proportion of c-Fos protein expression in VTA glutamatergic neurons from BY-R mice (n = 8 slices from 3 mice). Three times the experiment was repeated with similar results. **d,** Quantitative data of A-C. Control versus BY-S, P = 0.1761; Control versus BY-R, $P < 0.0001$; BY-S versus BY-R, $P = 8.1 \times 10^{-12}$. Scale bar: 600 μm. ****P<0.0001, ns: no significance. Data analyzed by Fisher's exact test. Statistical details are presented in Supplementary Table 5. Source data are provided as a Source Data file.

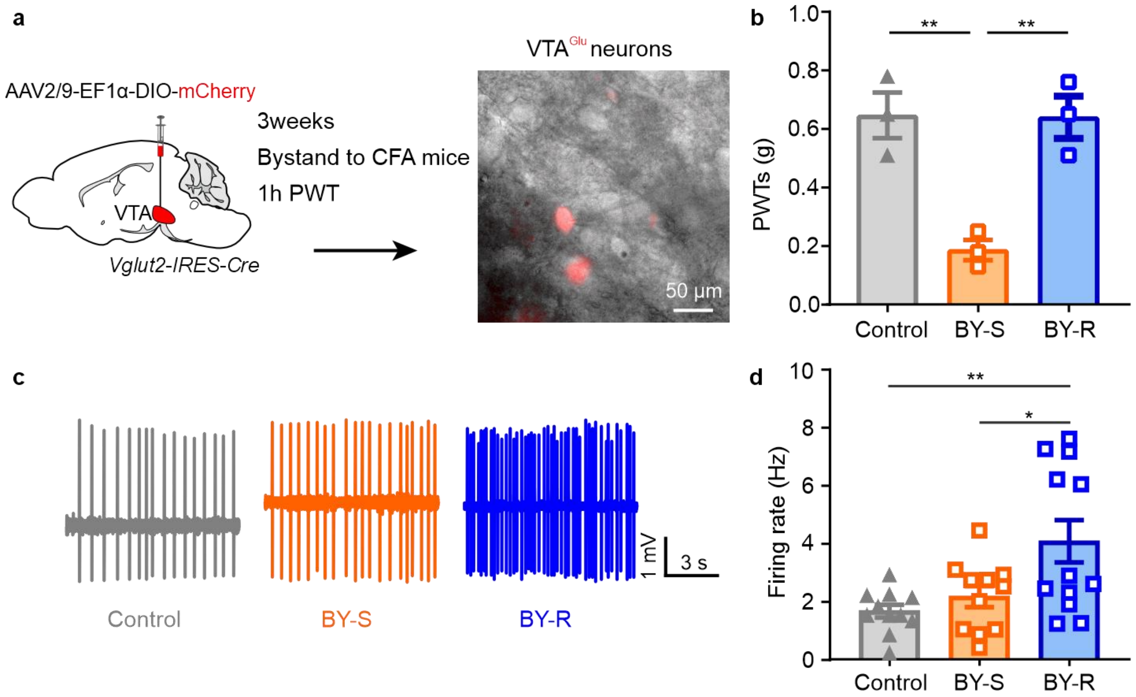

Supplementary Fig. 6 | Increased firing activity in VTA glutamatergic neurons of the BY-R mice. a, Schematic illustration depicting viral injection, experimental design, and representative image of mCherry-positive VTA vGlut2^{+} cells during patch clamp recording (Adapted from "The Mouse Brain in Stereotaxic Coordinates" by Paxinos and Franklin). Scale bar: 50 µm. **b**, PWTs ($n = 3$ mice per group). Control versus BY-S, $P = 0.0057$; BY-S versus BY-R, $P = 0.0061$. **c-d**, *In vitro* patch clamp sample traces (**c**) and firing rates (**d**) of VTA vGlut2^{+} neurons ($n = 11, 11, 12$ cells from 3, 3 and 3 mice). (**d**) Control versus BY-R, $P = 0.0056$; BY-S versus BY-R, $P = 0.0312$. *$P < 0.05$, **$P < 0.01$. Data were analyzed by one-way ANOVA with Tukey's multiple comparisons test. Statistical details are presented in Supplementary Table 5. Source data are provided as a Source Data file.

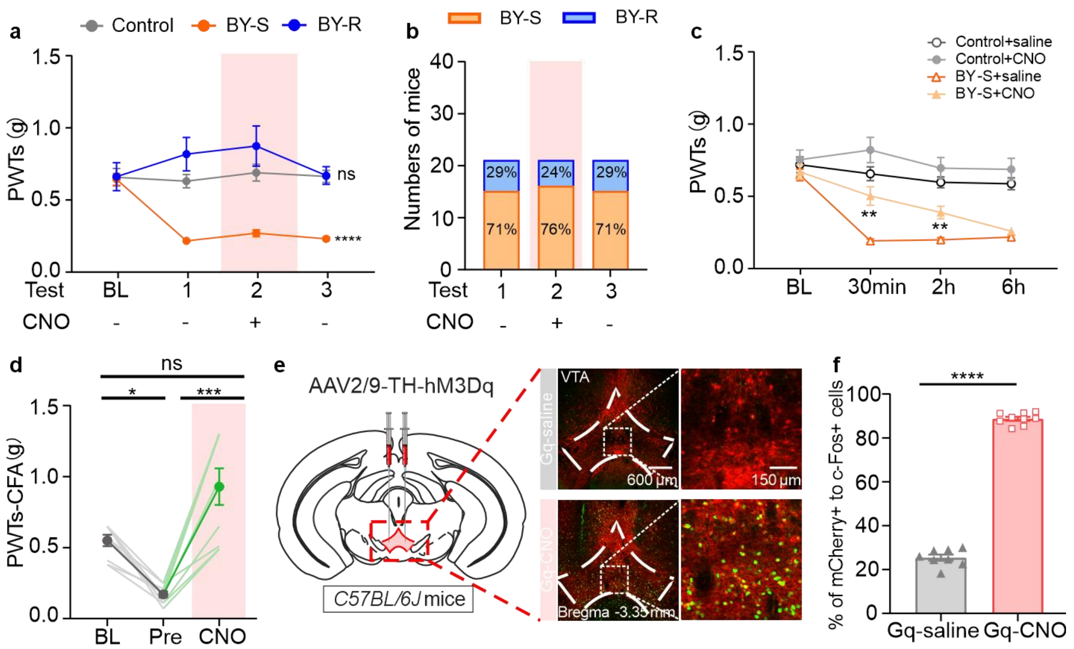

Supplementary Fig. 7 | Activation of VTA dopaminergic neurons does not affect the development of STA. a, PWTs across three repeats of social transfer of allodynia paradigm with or without chemogenetic activation of VTA dopaminergic neurons, subgroups of BY mice were identified following the first exposure to social contact with their CFA partners (*n* = 10, 14 and 5 mice). Control versus BY-S, P_{BL} = 0.9744, P_{T1} = 9.3 x 10^{-6}, P_{T2} = 7.0 x 10^{-5}, P_{T3} = 2.9 x 10^{-6}; Control versus BY-R, P_{BL} = 0.9993, P_{T1} = 0.3638, P_{T2} = 0.4906, P_{T3} = 0.9964. **b,** Percentages of BY-S and BY-R mice across the three repeats of paradigm. **c,** PWTs over different time points before and after CNO injection in BY-S mice (*n* = 11, 10, 14 and 13 mice). BY-S+saline versus BY-S+CNO, P_{BL} = 0.9871, P_{30min} = 0.0019, P_{2h} = 0.0047, P_{6h} = 0.1871. **d,** PWTs for CFA mice at baseline, before, and after CNO injection (*n* = 9 mice). BL versus Pre, *P* = 0.0201; Pre versus CNO, *P* > 0.9999; BL versus CNO, *P* = 0.0008. **e,** Schematic and representative immunofluorescent images illustrating c-Fos protein expression in mCherry-positive VTA dopaminergic neurons after saline and CNO treatment in C57BL/6J mice (Adapted from "The Mouse Brain in Stereotaxic Coordinates" by Paxinos and Franklin). Scale bar: 600 µm, 150 µm. The staining was repeated three times with similar results. **f,** Quantitative data showing the percentage of mCherry and c-Fos double stained cells out of c-Fos protein-positive cells (*n* = 8, 9 slices from 3 mice per group). $t_{(15)}$ = 39.3, *P* < 0.0001. The data are presented as the mean ± s.e.m. **P* < 0.05*,* ***P* < 0.01, ****P* < 0.001, *****P* < 0.0001, ns: no significance. Data analyzed by (**a, c**) Two-way RM ANOVA with Tukey's multiple comparisons test; (**b**) chi-square test; (**d**) two-sided Friedman test with Dunn's multiple comparisons test; or (**f**) unpaired two-sided *t*-test. Statistical details are presented in Supplementary Table 5. Source data are provided as a Source Data file.

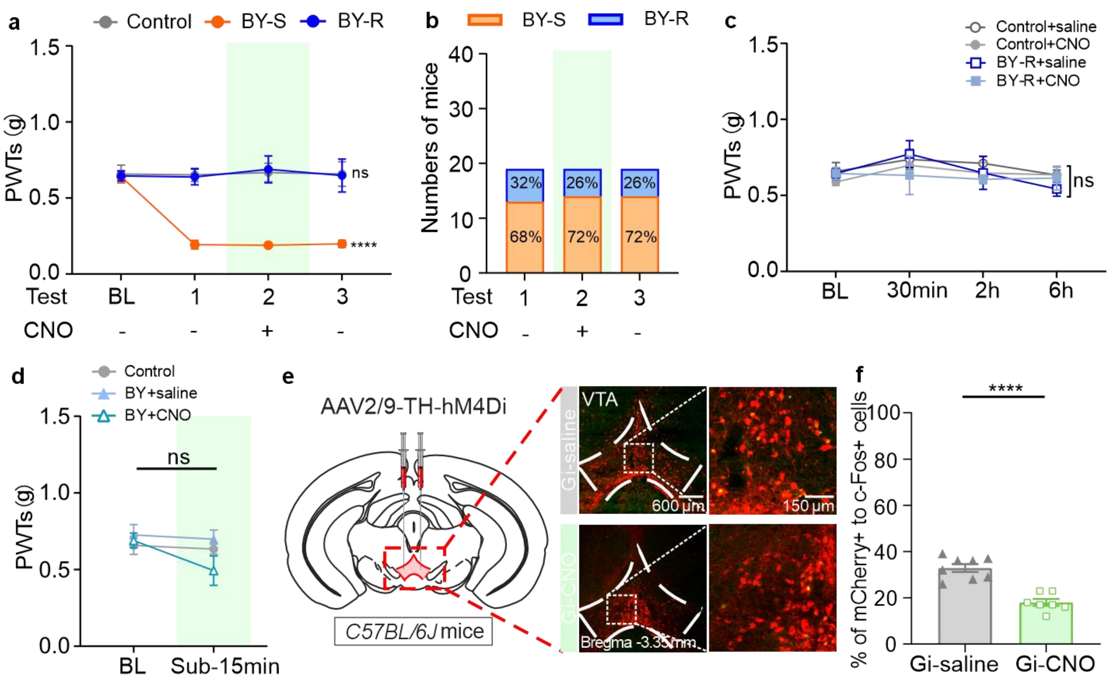

Supplementary Fig. 8 | Inhibition of VTA dopaminergic neurons is not required for the development and maintenance of STA. a, PWTs across three repeats of social transfer of allodynia paradigm with or without chemogenetic inhibition of VTA dopaminergic neurons, subgroups of bystander mice were identified following the first exposure to social contact with their CFA partners (*n* = 10, 13 and 6 mice). Control versus BY-S, P = 0.9679, = 4.5 x 10^{-7}, = 4.1 x 10^{-5}, = 0.0006; Control versus BY-R, P_{BL} = 0.9795, P_{T1} = 0.9780, P_{T2} = 0.9786, P_{T3} = 0.9899. **b,** Percentages of BY-S and BY-R mice across the three repeats of paradigm. **c,** PWTs over different time points before and after CNO injection in BY-R mice (*n* = 10, 10, 5 and 5 mice). BY-R+saline versus BY-R+CNO, $P_{BL} > 0.9999$, P_{30min} = 0.8007, P_{2h} = 0.9800, P_{6h} = 0.8603. **d,** PWTs after chemogenetic inhibition of VTA glutamatergic neurons during the sub-threshold paradigm (*n* = 10, 8 and 9 mice). **e,** Schematic and representative immunofluorescent images illustrating c-Fos protein expression in mCherry-positive VTA dopaminergic neurons after saline and CNO treatment in C57BL/6J mice. Scale bar: 600 µm, 150 µm. The staining was repeated three times with similar results (Adapted from "The Mouse Brain in Stereotaxic Coordinates" by Paxinos and Franklin). **f,** Quantitative data showing the percentage of mCherry and c-Fos double stained cells out of c-Fos protein-positive cells (*n* = 8, 7 from 3 mice per group). $t_{(13)}$ = 6.517, P = 2.0 x 10^{-5}. The data are presented as the mean ± s.e.m. *$P < 0.05$, **$P < 0.01$, ***$P < 0.001$, ****$P < 0.0001$, ns: no significance. Data analyzed by (**a, c, d**) Two-way RM ANOVA with Tukey's multiple comparisons test; (**b**) chi-square test; or (**f**) unpaired two-sided *t*-test. Statistical details are presented in Supplementary Table 5. Source data are provided as a Source Data file.

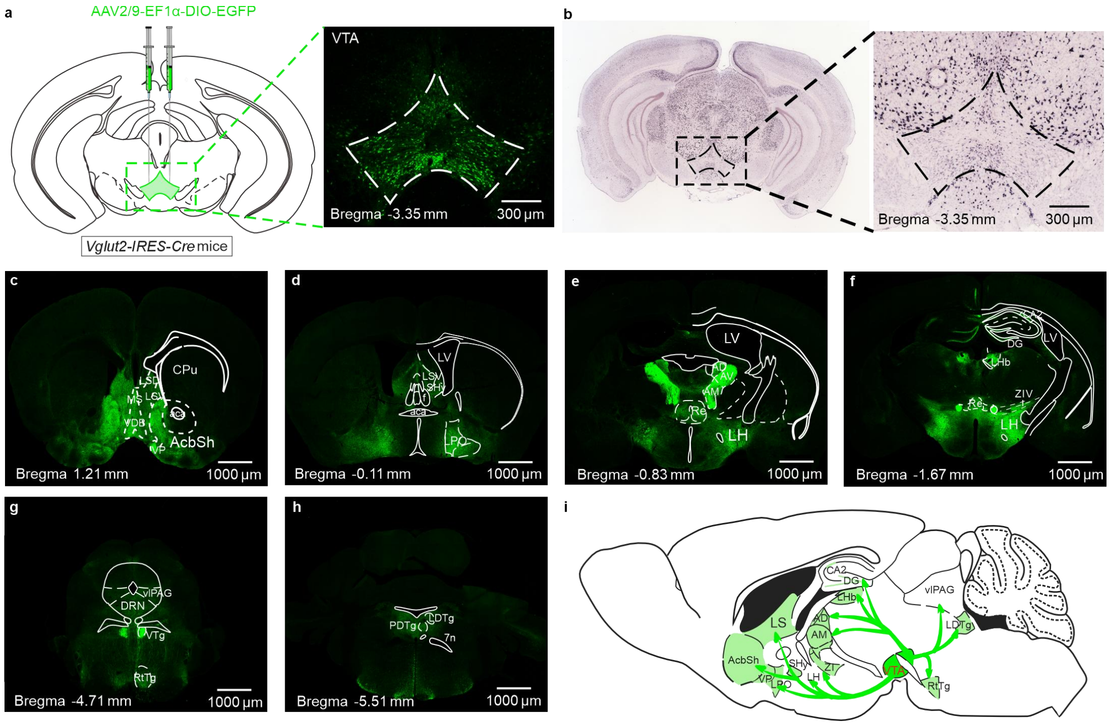

Supplementary Fig. 9 | Identification of the downstream brain regions of VTA glutamatergic projections in *Vglut2-IRES-Cre* mice. a, Schematic of viral injection (left) and representative neuronal EGFP expression in the VTA of *Vglut2-IRES-Cre* mice (right). Scale bar: 300 μm. **b,** A representative in situ hybridization image showing the *Slc17a6* gene (*Vglut2*) expression in the VTA (E. S. Lein *et al.*, 2007). **c-h,** Representative immunofluorescent images showing EGFP-labeled fibers in the indicated regions. LS, lateral septum; CPu, caudate putamen; NAc, nucleus accumbens; VDB, the nucleus of the vertical limb of the diagonal band; LPO, lateral preoptic area; SHy, septohypothalamic nucleus; AD, anterodorsal thalamic nucleus; AV, anteroventral thalamic nucleus; AM, anteromedial thalamic nucleus; Re, reuniens thalamic nucleus; LH, lateral hypothalamic area; CA2, field CA2 of the hippocampus; DG, dentate gyrus; LHb, lateral habenular nucleus; ZI, zona incerta; vlPAG, ventrolateral periaqueductal gray; DRN, dorsal raphe nucleus; RtTg, reticulotegmental nucleus of the pons; DTgP, dorsal tegmental nucleus, pericentral part. Scale bar: 1000 μm. **i,** Schematic showing the VTA glutamatergic efferents. The staining was repeated twice with similar results. Figures adapted from "The Mouse Brain in Stereotaxic Coordinates" by Paxinos and Franklin.

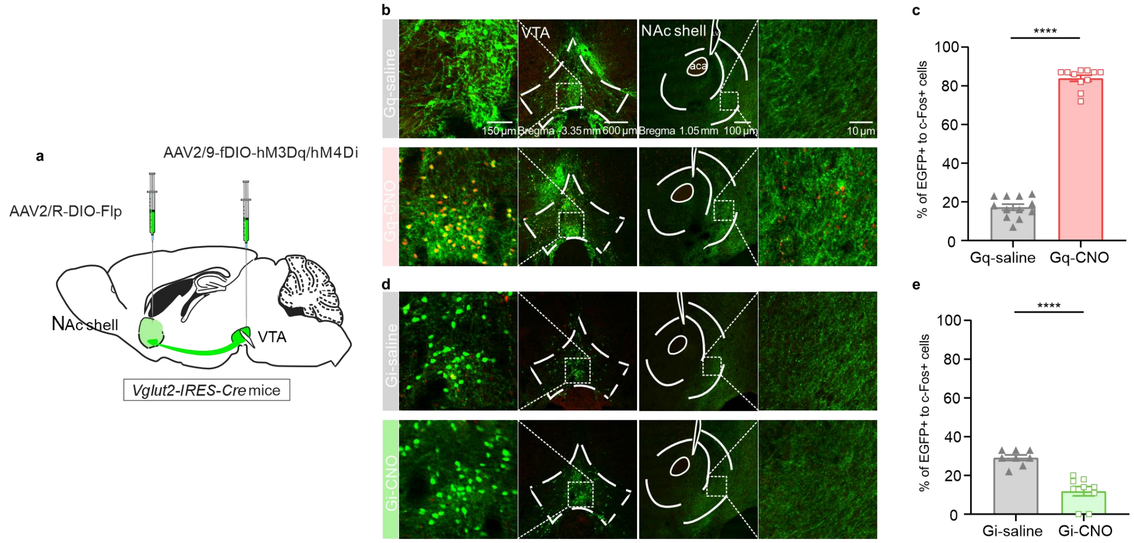

Supplementary Fig. 10 | Chemogenetic manipulation of the VTA→NAc shell glutamatergic circuit bidirectionally regulate cell type-specific c-Fos protein expression. a, Schematic showing viral injection surgeries (Adapted from "The Mouse Brain in Stereotaxic Coordinates" by Paxinos and Franklin). **b,** Representative immunofluorescent images illustrating c-Fos protein expression in EGFP-positive VTA glutamatergic neurons after saline or CNO treatment, and representative EGFP positive terminals in the NAc shell in *Vglut2-IRES-Cre* mice expressing hM3Dq in their VTA→NAc shell projecting glutamatergic neuons. Scale bar: 600 μm, 150 μm, 300 μm and 40 μm. The staining was repeated four times with similar results. **c,** Quantitative data of c-Fos protein expression in EYFP-positive VTA glutamatergic neurons (n = 12, 11 slices from 4 mice per group). $U = 0$, $P = 1.5 \times 10^{-6}$. **d,** Representative immunofluorescent images illustrating c-Fos protein expression in EGFP-positive VTA glutamatergic neurons after saline or CNO treatment, and representative EGFP positive terminals in the NAc shell in *Vglut2-IRES-Cre* mice expressing hM4Di in their VTA→NAc shell projecting glutamatergic neuons. Three times the experiment was repeated with similar results. **e,** Quantitative data of c-Fos protein expression in EYFP-positive VTA glutamatergic neurons (n = 8, 9 from 3 mice per group). $t_{(15)} = 5.935$, $P = 2.7 \times 10^{-5}$. The data are expressed as the mean ± s.e.m. ****$P < 0.0001$. Data analyzed by (**c**) Mann-Whitney test; or (**e**) unpaired two-sided t-test. Statistical details are presented in Supplementary Table 5. Source data are provided as a Source Data file.

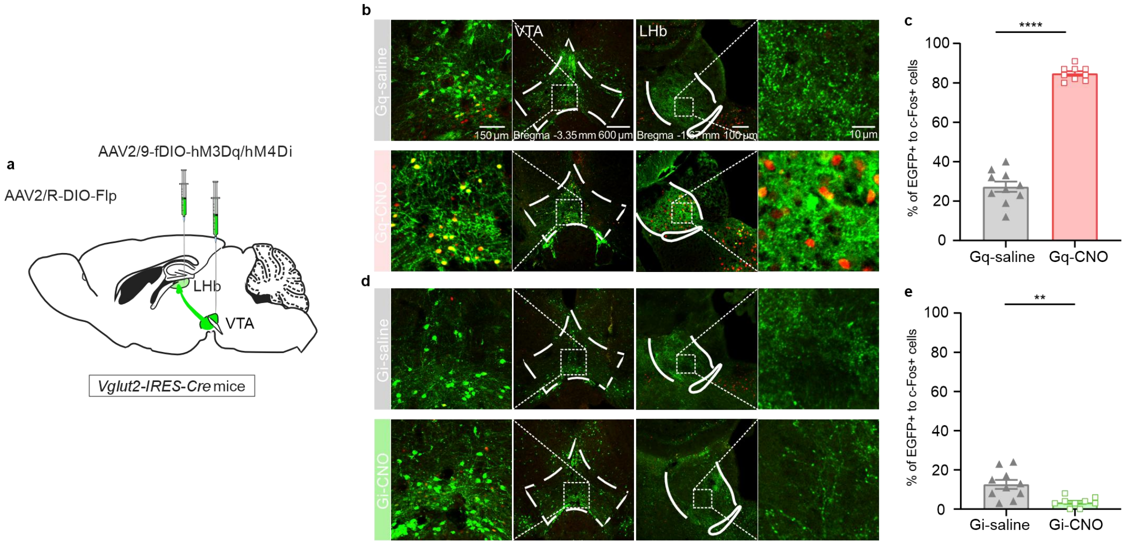

Supplementary Fig. 11 | Chemogenetic manipulation of the VTA→LHb glutamatergic circuit bidirectionally regulate cell type-specific c-Fos protein expression. a, Schematic showing viral injection surgeries (Adapted from "The Mouse Brain in Stereotaxic Coordinates" by Paxinos and Franklin). **b,** Representative immunofluorescent images illustrating c-Fos protein expression in EGFP-positive VTA glutamatergic neurons after saline or CNO treatment, and representative EGFP positive terminals in the NAc shell in *Vglut2-IRES-Cre* mice expressing hM3Dq in their VTA→LHb projecting glutamatergic neuons. Scale bar: 600 µm, 150 µm, 100 µm and 10 µm. **c,** Quantitative data of c-Fos protein expression in EYFP-positive VTA glutamatergic neurons (n = 10, 9 slices from 3 and 4 mice). $t_{(17)} = 19.15$, $P = 6.1 \times 10^{-13}$. **d,** Representative immunofluorescent images illustrating c-Fos protein expression in EGFP-positive VTA glutamatergic neurons after saline or CNO treatment, and representative EGFP positive terminals in the LHb in *Vglut2-IRES-Cre* mice expressing hM4Di in their VTA→LHb projecting glutamatergic neuons. **e,** Quantitative data of c-Fos protein expression in EYFP-positive VTA glutamatergic neurons (n = 8, 9 slices from 3 mice per group). $t_{(17)} = 3.580$, $P = 0.0023$. The staining was repeated three times with similar results. The data are expressed as the mean ± s.e.m. ****$P < 0.0001$. Data analyzed by (**c, e**) unpaired two-sided *t*-test. Statistical details are presented in Supplementary Table 5. Source data are provided as a Source Data file.

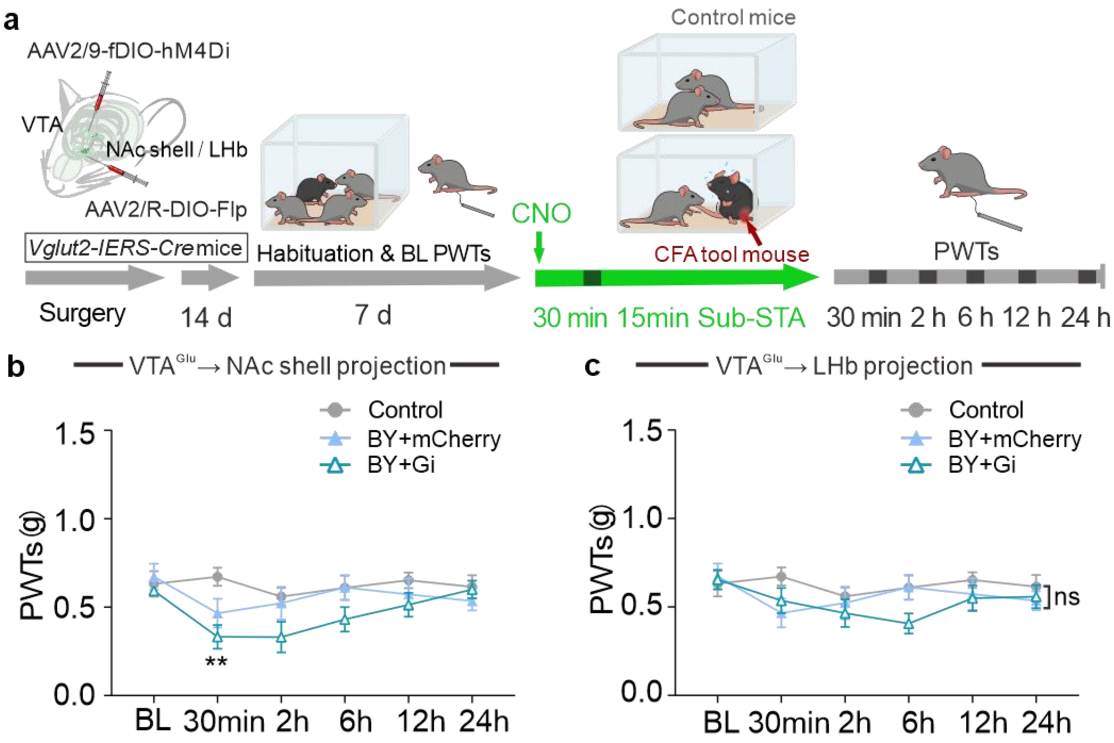

Supplementary Fig. 12 | Chemogenetic activation of the VTA→NAc shell glutamatergic circuit, but not the VTA → LHb circuit, promotes the development of STA in mice undergone the subthreshold paradigm. a, Experimental timeline. **b,** PWTs after chemogenetic inhibition of VTA→NAc shell-projecting glutamatergic neurons during the sub-threshold social contact (n = 9, 8 and 8 mice). Control versus BY+Gi, P_{BL} = 0.8625, P_{30min} = 0.0034, P_{2h} = 0.0981, P_{6h} = 0.1897, P_{12h} = 0.2391, P_{24h} = 0.9820. **c,** PWTs after chemogenetic inhibition of VTA→LHb shell-projecting glutamatergic neurons in a 15 min of sub-threshold paradigm (n = 10, 8 and 8 mice). The data are expressed as the mean ± s.e.m. $^{**}P < 0.01$, ns: no significance. Data analyzed by (**p, q**) Two-way RM ANOVA with Tukey's multiple comparisons test. Statistical details are presented in Supplementary Table 5. Source data are provided as a Source Data file.

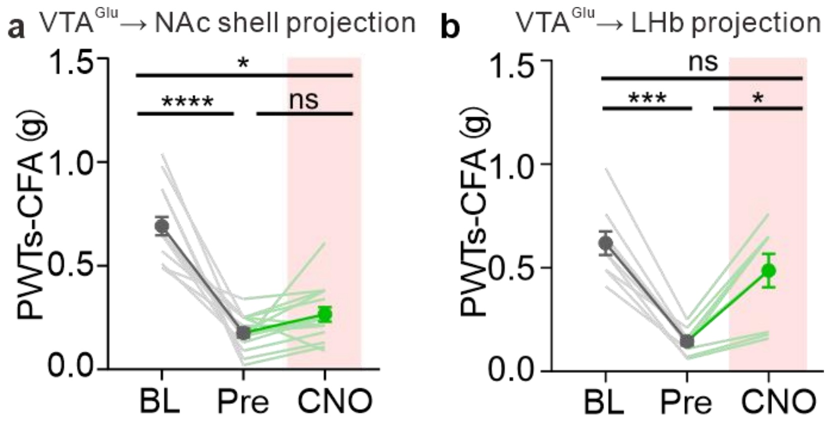

Supplementary Fig. 13 | Chemogenetic activation of the VTA→LHb glutamatergic circuit, but not the VTA→NAc shell circuit, alleviates mechanical allodynia in CFA mice. a, PWTs in CFA mice with and without chemogenetic activation of VTA→NAc shell glutamatergic projection (*n* = 15 mice). BL versus Pre, $P = 3.9 \times 10^{-6}$; Pre versus CNO, *P* = 0.0140; BL versus CNO, *P* = 0.1338. **b,** PWTs in CFA mice with and without chemogenetic activation of the VTA→LHb glutamatergic projection (*n* = 9 mice). BL versus Pre, *P* = 0.0008; Pre versus CNO, *P* > 0.9999; BL versus CNO, *P* = 0.0201. The data are expressed as the mean ± s.e.m. ***P* < 0.01, ****P* < 0.001, *****P* < 0.0001, ns: no significance. Data analyzed by (**a, b**) Friedman test with Dunn's multiple comparisons test. Statistical details are presented in Supplementary Table 5. Source data are provided as a Source Data file.

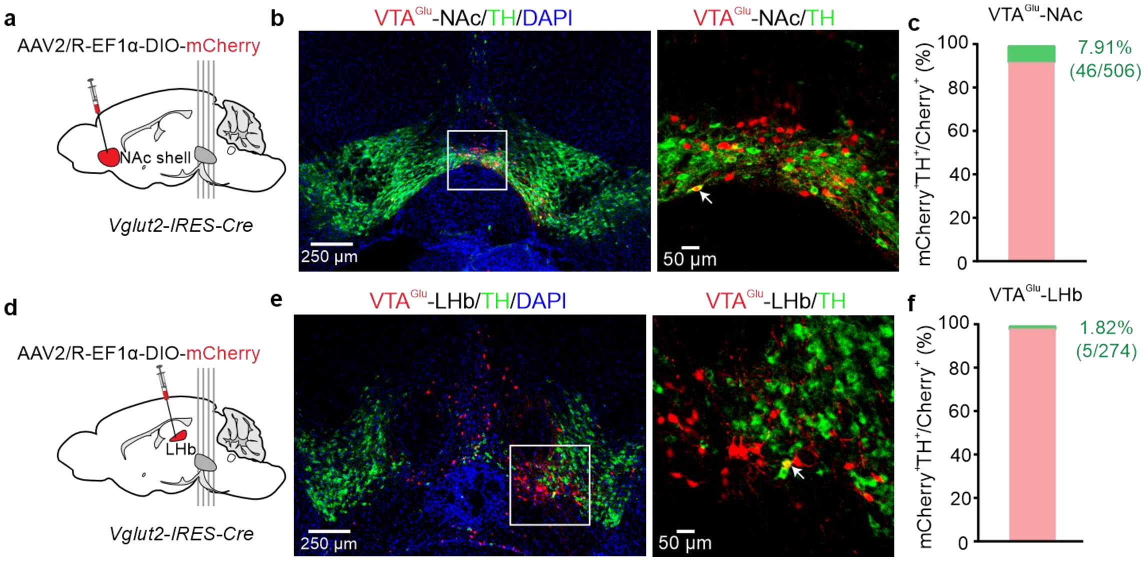

Supplementary Fig. 14 | Co-expression of TH in VTA projecting glutamatergic neurons. a, Schematic for retrograde AAV-DIO-mCherry injection into the NAc shell of *Vglut2-IRES-Cre* mice (Adapted from "The Mouse Brain in Stereotaxic Coordinates" by Paxinos and Franklin). **b and c,** Representative immunofluorescent images and quantitative data for co-expression of TH in VTA→NAc shell projecting gluatmatergic neurons. Scale bar: 250 μm, 50 μm. **d,** Schematic for retrograde AAV-DIO-mCherry injection into the LHb of *Vglut2-IRES-Cre* mice (Adapted from "The Mouse Brain in Stereotaxic Coordinates" by Paxinos and Franklin). **e and f,** Representative immunofluorescent images and quantitative data for co-expression of TH in VTA→LHb projecting gluatmatergic neurons. Scale bar: 250 μm and 50 μm. White arrows indicate VTA projecting glutamatergic neurons co-expressing TH. Three times the experiment was repeated with similar results.

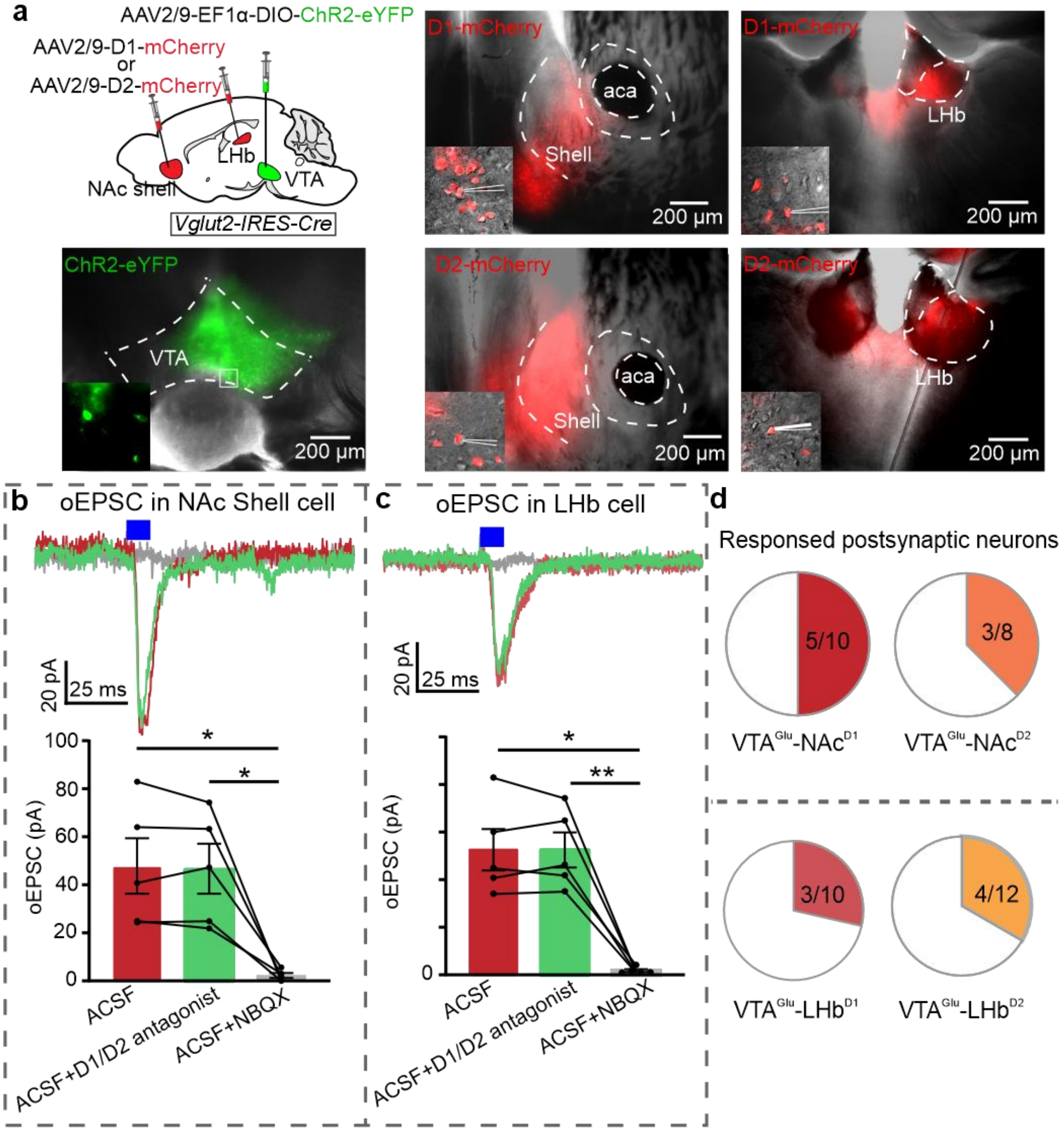

Supplementary Fig. 15 | Postsynaptic characteristics of VTA glutamatergic neurons in NAc shell and LHb. a, Schematic for anterograde AAV-DIO-ChR2-eYFP injection into the VTA and AAV-D1-mCherry or AAV-D2-mCherry into the NAc shell or LHb of *Vglut2-IRES-Cre* mice (Adapted from "The Mouse Brain in Stereotaxic Coordinates" by Paxinos and Franklin). Representative immunofluorescent images of virus expression in VTA, NAc and LHb were shown. Scale bar: 200 μm. **b,** Representative responsive traces and quantitative data for optogenetic stimulation of VTA gluatmatergic terminals in NAc shell neurons in the presence of ACSF, D1/D2 antagonist and NBQX, respectively (n = 5 cells from 4 mice per grouop). ACSF versus ACSF+NBQX, P = 0.0111; ACSF+D1/D2 antagonist versus ACSF+NBQX, P = 0.0131. **c,** Representative responsive traces and quantitative data for optogenetic stimulation of VTA gluatmatergic terminals in LHb neurons in the presence of ACSF, D1/D2 antagonist and NBQX, respectively (n = 5 cells from 4 mice per group). ACSF versus ACSF+NBQX, P = 0.0134;

ACSF+D1/D2 antagonist versus ACSF+NBQX, $P = 0.0075$. **d,** Summary data of responsed postsynaptic cells during optogenetic stimulation of VTA gluatmatergic terminals in NAc shell and LHb. Scale bar: 200 µm. The staining was repeated four times with similar results. The data are expressed as the mean ± s.e.m. $*P < 0.05$, $**P < 0.01$. Data analyzed by (**b, c**) one-way ANOVA with Tukey's multiple comparisons test. Statistical details are presented in Supplementary Table 5. Source data are provided as a Source Data file.

Supplementary Table 1. Extended statistical information for Fig. 1c.

	Control	BY-S	BY-R
Sample Size	111	137	72
25% Percentile	0.5	0.13	0.57
Median	0.61	0.18	0.65
75% Percentile	0.76	0.25	0.78
Mean	0.65	0.20	0.7
Standard Deviation	0.20	0.09	0.17
Standard Error	0.02	0.01	0.02
Lower 95% CI of Mean	0.61	0.18	0.66
Upper 95% CI of Mean	0.68	0.21	0.74

Supplementary Table 2. Extended statistical information for Fig. 1e.

	Control	BY-S	BY-R
Sample Size	111	146	63
25% Percentile	78.46	24.14	87.69
Median	100	32.48	100
75% Percentile	125	47.78	132.65
Mean	108.11	34.95	113.62
Standard Deviation	43.39	16.60	35.72
Standard Error	4.12	1.37	4.50
Lower 95% CI of Mean	99.94	32.24	104.63
Upper 95% CI of Mean	116.27	37.67	122.62

Supplementary Table 3. Extended statistical information for Extended Data Fig. 2a.

	Control	BY-S	BY-R
Sample Size	10	15	5
25% Percentile	0.55	0.16	0.49
Median	0.65	0.21	0.51
75% Percentile	0.79	0.25	0.54
Mean	0.67	0.21	0.51
Standard Deviation	0.17	0.06	0.03
Standard Error	0.05	0.01	0.01
Lower 95% CI of Mean	0.55	0.18	0.47
Upper 95% CI of Mean	0.79	0.24	0.55

Supplementary Table 4. Extended statistical information for Extended Data Fig. 2B.

	Control	BY-S	BY-R
Sample Size	10	16	4
25% Percentile	72.62	21.46	76.15
Median	91.83	25.87	78.46
75% Percentile	138.27	38.03	85.38
Mean	103.46	30.60	80.00
Standard Deviation	38.45	11.68	5.33
Standard Error	12.16	2.92	2.66
Lower 95% CI of Mean	75.96	24.37	71.52
Upper 95% CI of Mean	130.97	36.82	88.48

Supplementary Table 5. Extended statistical information for Fig. 1-6, Extended Data Fig. 1-15.

Figure	n/group	Primary statistic	*Post-hoc* test	Comparison	*p* value	Notation	*F/t* statistic
**Fig. 1c**	Control, n = 111 mice<br>BY-S, n = 137 mice<br>BY-R, n = 72 mice	*Kruskal-Wallis test*	*Dunn's*	Summary	<0.0001	****	H = 236.5
				Control vs. BY-S	<0.0001	****	
				Control vs. BY-R	0.4058	ns	
				BY-S vs. BY-R	<0.0001	****	
**Fig. 1e**	Control, n = 111 mice<br>BY-S, n = 146 mice<br>BY-R, n = 63 mice	*Kruskal-Wallis test*	*Dunn's*	Summary	<0.0001	****	H = 234.3
				Control vs. BY-S	<0.0001	****	
				Control vs. BY-R	0.9796	ns	
				BY-S vs. BY-R	<0.0001	****	
**Fig. 1g**	1h, n = 40 mice<br>2h, n = 40 mice	*chi-square test*			0.7483	ns	$\chi^2 = 0.5800$
	1h, BY-S n = 26 mice<br>1h, BY-R n = 11 mice<br>1h, Erratic n = 3 mice						
	2h, BY-S n = 24 mice<br>2h, BY-R n = 11 mice<br>2h, Erratic n = 5 mice						
**Fig. 1i**	Control, n = 18 mice<br>BY-S, n = 13 mice<br>BY-R, n = 6 mice	*Two-way RM ANOVA*	*Sidak's*	Group × time interaction	0.4879	ns	F (2, 34) = 0.7330
				Left vs. Right in Control	0.5929	ns	
				Left vs. Right in BY-S	0.9995	ns	
				Left vs. Right in BY-R	0.8706	ns	

**Supplementary Fig. 1**	Control, n = 10 mice<br>BY, n = 10 mice	*Two-way ANOVA*	*Sidak's*	Group × time interaction	3.5×10^{-6}	****	F (4, 72) = 9.418
				Control vs. BY at Baseline	0.7615	ns	
				Control vs. BY at 0h	0.0243	*	
				Control vs. BY at 4h	0.0089	**	
				Control vs. BY at 6h	0.0481	*	
				Control vs. BY at 24h	>0.9999	ns	
**Supplementary Fig. 2a**	Control, n = 10 mice<br>BY-S, n = 15 mice<br>BY-R, n = 5 mice	*One-way ANOVA*	*Tukey's*	Main effect of group	2.3×10^{-11}	****	F (2, 26) = 72.52
				Control vs. BY-S	2.9×10^{-11}	****	
				Control vs. BY-R	0.0530	ns	
				BY-S vs. BY-R	6.8×10^{-7}	****	
**Supplementary Fig. 2b**	Control, n = 10 mice<br>BY-S, n = 16 mice<br>BY-R, n = 4 mice	*One-way ANOVA*	*Tukey's*	Main effect of group	1.4×10^{-7}	****	F (2, 26) = 34.11
				Control vs. BY-S	1.2×10^{-7}	****	
				Control vs. BY-R	0.3917	ns	
				BY-S vs. BY-R	0.0004	***	
**Supplementary Fig. 2c**	male, n = 20 mice<br>female, n = 20 mice	*chi-square test*			0.7483	ns	$\chi2 = 0.5873$
	male, BY-S n = 13 mice<br>male, BY-R n = 5 mice<br>male, Erratic n = 2 mice						
	female, BY-S n = 15 mice<br>female, BY-R n = 4 mice<br>female, Erratic n = 1 mice						
**Supplementary**	Control, n = 18 mice	*Kruskal-Wallis test*	*Dunn's*	Summary	0.1921	ns	H = 4.738

**Fig. 3b**	BY-S, n = 13 mice<br>BY-R, n = 7 mice<br>CFA, n = 20 mice						
				Control vs. BY-S	>0.9999	ns	
				Control vs. BY-R	>0.9999	ns	
				Control vs. CFA	>0.9999	ns	
				BY-S vs. BY-R	>0.9999	ns	
				BY-S vs. CFA	0.2287	ns	
				BY-R vs. CFA	>0.9999	ns	
**Supplementary Fig. 3c**	Control, n = 18 mice<br>BY-S, n = 13 mice<br>BY-R, n = 7 mice<br>CFA, n = 20 mice	*Two-way RM ANOVA*	*Tukey's*	Group × time interaction	0.2559	ns	$F(3, 54) = 1.389$
				Control vs. BY-S with normal mice	0.8914	ns	
				Control vs. BY-R with normal mice	0.9826	ns	
				Control vs. CFA with normal mice	0.8851	ns	
				BY-S vs. BY-R with normal mice	0.8018	ns	
				BY-S vs. CFA with normal mice	0.5056	ns	
				BY-R vs. CFA with normal mice	0.9984	ns	
				Control vs. BY-S with CFA mice	0.9985	ns	
				Control vs. BY-R with CFA mice	0.9271	ns	
				Control vs. CFA with CFA mice	0.8786	ns	
				BY-S vs. BY-R with CFA mice	0.9676	ns	
				BY-S vs. CFA with CFA mice	0.8335	ns	
				BY-R vs. CFA with CFA mice	0.6438	ns	
**Supplementary Fig. 3d**	Control, n = 18 mice<br>BY, n = 20 mice<br>CFA, n = 20 mice	*Two-way RM ANOVA*	*Tukey's*	Group × time interaction	0.4725	ns	$F(2, 55) = 0.7601$

				Control vs. BY with normal mice	0.5609	ns	
				Control vs. CFA with normal mice	>0.9999	ns	
				BY vs. CFA with normal mice	0.5375	ns	
				Control vs. BY with CFA mice	0.9982	ns	
				Control vs. CFA with CFA mice	0.9540	ns	
				BY vs. CFA with CFA mice	0.9686	ns	
**Supplementary Fig. 3e**	Control, n = 18 mice<br>BY-S, n = 13 mice<br>BY-R, n = 7 mice<br>CFA, n = 20 mice	*Two-way RM ANOVA*	*Tukey's*	Group × time interaction	0.3901	ns	F (3, 54) = 1.022
				Control vs. BY-S with normal mice	0.4782	ns	
				Control vs. BY-R with normal mice	>0.9999	ns	
				Control vs. CFA with normal mice	>0.9999	ns	
				BY-S vs. BY-R with normal mice	0.6520	ns	
				BY-S vs. CFA with normal mice	0.4542	ns	
				BY-R vs. CFA with normal mice	>0.9999	ns	
				Control vs. BY-S with CFA mice	>0.9999	ns	
				Control vs. BY-R with CFA mice	0.9981	ns	
				Control vs. CFA with CFA mice	0.9913	ns	
				BY-S vs. BY-R with CFA mice	0.9974	ns	
				BY-S vs. CFA with CFA mice	0.9904	ns	
				BY-R vs. CFA with CFA mice	>0.9999	ns	
**Supplementary Fig. 4b**	Control, n = 6 mice<br>BY-S, n = 8 mice<br>BY-R, n = 6 mice	*Kruskal-Wallis test*	*Dunn's*	Summary	0.0283	*	H = 6.690
				Control vs. BY-S	0.0397	*	
				Control vs. BY-R	0.1529	ns	

				BY-S vs. BY-R	>0.9999	ns	
**Supplementary Fig. 4c**	Control, n = 6 mice<br>BY-S, n = 8 mice<br>BY-R, n = 6 mice	*Kruskal-Wallis test*	*Dunn's*	Summary	0.0002	***	H = 12.45
				Control vs. BY-S	0.0020	**	
				Control vs. BY-R	0.0288	*	
				BY-S vs. BY-R	>0.9999	ns	
**Supplementary Fig. 4d**	Control, n = 6 mice<br>BY-S, n = 8 mice<br>BY-R, n = 6 mice	*Kruskal-Wallis test*	*Dunn's*	Summary	0.0002	***	H = 12.41
				Control vs. BY-S	0.0020	**	
				Control vs. BY-R	0.0291	*	
				BY-S vs. BY-R	>0.9999	ns	
**Supplementary Fig. 4e**	Control, n = 6 mice<br>BY-S, n = 8 mice<br>BY-R, n = 6 mice	*Kruskal-Wallis test*	*Dunn's*	Summary	0.00009	****	H = 13.21
				Control vs. BY-S	0.0010	***	
				Control vs. BY-R	0.0524	ns	
				BY-S vs. BY-R	0.8754	ns	
**Supplementary Fig. 4f**	Control, n = 6 mice<br>BY-S, n = 8 mice<br>BY-R, n = 6 mice	*Two-way RM ANOVA*	*Tukey's*	Group × time interaction	0.5337	ns	F (6, 51) = 0.8555
				Control vs. BY-S at 0-15	0.0489	*	
				Control vs. BY-R at 0-15	0.1211	ns	
				BY-S vs. BY-R at 0-15	0.7792	ns	

				Control vs. BY-S at 15-30	0.9928	ns	
				Control vs. BY-R at 15-30	0.8690	ns	
				BY-S vs. BY-R at 15-30	0.6340	ns	
				Control vs. BY-S at 30-45	0.4251	ns	
				Control vs. BY-R at 30-45	0.5386	ns	
				BY-S vs. BY-R at 30-45	0.9577	ns	
				Control vs. BY-S at 45-60	0.0509	ns	
				Control vs. BY-R at 45-60	0.0950	ns	
				BY-S vs. BY-R at 45-60	0.6921	ns	
**Supplementary Fig. 4g**	Control, n = 6 mice<br>BY-S, n = 8 mice<br>BY-R, n = 6 mice	*Two-way RM ANOVA*	*Tukey's*	Group × time interaction	0.0558	ns	F (6, 51) = 2.222
				Control vs. BY-S at 0-15	0.0186	*	
				Control vs. BY-R at 0-15	0.1764	ns	
				BY-S vs. BY-R at 0-15	0.7800	ns	
				Control vs. BY-S at 15-30	0.0668	ns	
				Control vs. BY-R at 15-30	0.2617	ns	
				BY-S vs. BY-R at 15-30	0.6872	ns	
				Control vs. BY-S at 30-45	0.0221	*	
				Control vs. BY-R at 30-45	0.2445	ns	
				BY-S vs. BY-R at 30-45	0.6385	ns	
				Control vs. BY-S at 45-60	0.0934	ns	
				Control vs. BY-R at 45-60	0.0492	*	
				BY-S vs. BY-R at 45-60	0.2450	ns	
**Supplementary Fig. 4h**	Control, n = 6 mice<br>BY-S, n = 8 mice<br>BY-R, n = 6 mice	*Two-way RM ANOVA*	*Tukey's*	Group × time interaction	0.2954	ns	F (6, 51) = 1.253
				Control vs. BY-S at 0-15	0.0153	*	

				Control vs. BY-R at 0-15	0.0105	*	
				BY-S vs. BY-R at 0-15	>0.9999	ns	
				Control vs. BY-S at 15-30	0.0015	**	
				Control vs. BY-R at 15-30	0.0373	*	
				BY-S vs. BY-R at 15-30	0.9964	ns	
				Control vs. BY-S at 30-45	0.0264	*	
				Control vs. BY-R at 30-45	0.2082	ns	
				BY-S vs. BY-R at 30-45	0.9869	ns	
				Control vs. BY-S at 45-60	0.0010	**	
				Control vs. BY-R at 45-60	0.0330	*	
				BY-S vs. BY-R at 45-60	0.5590	ns	
**Supplementary Fig. 4i**	Control, n = 6 mice<br>BY-S, n = 8 mice<br>BY-R, n = 6 mice	*Two-way RM ANOVA*	*Tukey's*	Group × time interaction	0.0308	*	F (6, 51) = 2.552
				Control vs. BY-S at 0-15	0.0014	**	
				Control vs. BY-R at 0-15	0.1365	ns	
				BY-S vs. BY-R at 0-15	0.5465	ns	
				Control vs. BY-S at 15-30	0.0189	*	
				Control vs. BY-R at 15-30	0.1621	ns	
				BY-S vs. BY-R at 15-30	0.8853	ns	
				Control vs. BY-S at 30-45	0.0001	***	
				Control vs. BY-R at 30-45	0.0544	ns	
				BY-S vs. BY-R at 30-45	0.1214	ns	
				Control vs. BY-S at 45-60	0.0164	*	
				Control vs. BY-R at 45-60	0.0199	*	
				BY-S vs. BY-R at 45-60	0.1885	ns	
**Fig. 2b**	Control, n = 35 mice<br>BY-S, n = 26 mice	*Kruskal-Wallis test*	*Dunn's*	Summary	1.0×10^{-11}	****	H = 50.57

	BY-R, n = 12 mice						
				Control vs. BY-S	8.2 x 10^{-10}	****	
				Control vs. BY-R	0.9450	ns	
				BY-S vs. BY-R	4.9 x 10^{-8}	****	
**Fig. 2c** **ACC**	Control, n = 19 mice BY-S, n = 20 mice BY-R, n = 23 mice	*Kruskal-Wallis test*	*Dunn's*	Summary	0.0106	*	H = 9.090
				Control vs. BY-S	0.0078	**	
				Control vs. BY-R	0.4076	ns	
				BY-S vs. BY-R	0.3004	ns	
**Fig. 2c** **NAc**	Control, n = 22 mice BY-S, n = 13 mice BY-R, n = 15 mice	*One-way ANOVA*	*Holm-Sidak's*	Main effect of group	0.0006	***	F (2, 47) = 8.723
				Control vs. BY-S	0.0005	***	
				Control vs. BY-R	0.3700	ns	
				BY-S vs. BY-R	0.0087	**	
**Fig. 2c** **NAc core**	Control, n = 22 mice BY-S, n = 13 mice BY-R, n = 15 mice	*One-way ANOVA*	*Holm-Sidak's*	Main effect of group	0.0001	***	F (2, 47) = 10.89
				Control vs. BY-S	0.0001	***	
				Control vs. BY-R	0.4640	ns	
				BY-S vs. BY-R	0.0019	**	
**Fig. 2c** **NAc shell**	Control, n = 22 mice BY-S, n = 13 mice BY-R, n = 15 mice	*One-way ANOVA*	*Holm-Sidak's*	Main effect of group	0.0299	*	F (2, 47) = 3.787
				Control vs. BY-S	0.0286	*	
				Control vs. BY-R	0.9307	ns	
				BY-S vs. BY-R	0.0944	ns	

**Fig. 2c**<br>**PVT**	Control, n = 15 mice<br>BY-S, n = 16 mice<br>BY-R, n = 15 mice	*One-way ANOVA*	*Holm-Sidak's*	Main effect of group	0.0012	**	F (2, 43) = 7.947
				Control vs. BY-S	0.0126	*	
				Control vs. BY-R	0.0015	**	
				BY-S vs. BY-R	0.8046	ns	
**Fig. 2c**<br>**BLA**	Control, n = 23 mice<br>BY-S, n = 19 mice<br>BY-R, n = 22 mice	*One-way ANOVA*	*Holm-Sidak's*	Main effect of group	0.0042	**	F (2, 61) = 5.982
				Control vs. BY-S	0.0100	*	
				Control vs. BY-R	0.9998	ns	
				BY-S vs. BY-R	0.0100	*	
**Fig. 2c**<br>**CeA**	Control, n = 23 mice<br>BY-S, n = 20 mice<br>BY-R, n = 22 mice	*Kruskal-Wallis test*	*Dunn's*	Summary	0.0005	***	H = 15.36
				Control vs. BY-S	0.0079	**	
				Control vs. BY-R	>0.9999	ns	
				BY-S vs. BY-R	0.0006	***	
**Fig. 2c**<br>**LHb**	Control, n = 21 mice<br>BY-S, n = 23 mice<br>BY-R, n = 20 mice	*One-way ANOVA*	*Holm-Sidak's*	Main effect of group	0.0205	*	F (2, 61) = 4.143
				Control vs. BY-S	0.0695	ns	
				Control vs. BY-R	0.0310	*	
				BY-S vs. BY-R	0.9680	ns	
**Fig. 2c**<br>**MD**	Control, n = 20 mice<br>BY-S, n = 22 mice<br>BY-R, n = 19 mice	*Kruskal-Wallis test*	*Dunn's*	Summary	0.0445	*	H = 6.226
				Control vs. BY-S	0.0684	ns	

				Control vs. BY-R	0.1268	ns	
				BY-S vs. BY-R	>0.9999	ns	
**Fig. 2c**<br>**Re**	Control, n = 22 mice<br>BY-S, n = 22 mice<br>BY-R, n = 16 mice	*Kruskal-Wallis test*	*Dunn's*	Summary	1.9×10^{-5}	****	H = 21.74
				Control vs. BY-S	2.5×10^{-5}	****	
				Control vs. BY-R	0.0034	**	
				BY-S vs. BY-R	>0.9999	ns	
**Fig. 2c**<br>**ZI**	Control, n = 23 mice<br>BY-S, n = 21 mice<br>BY-R, n = 16 mice	*One-way ANOVA*	*Holm-Sidak's*	Main effect of group	0.4986	ns	F (2, 57) = 0.7045
				Control vs. BY-S	0.5633	ns	
				Control vs. BY-R	0.9101	ns	
				BY-S vs. BY-R	0.9477	ns	
**Fig. 2c**<br>**LH**	Control, n = 21 mice<br>BY-S, n = 16 mice<br>BY-R, n = 15 mice	*Kruskal-Wallis test*	*Dunn's*	Summary	0.0011	**	H = 13.65
				Control vs. BY-S	0.0011	**	
				Control vs. BY-R	0.0538	ns	
				BY-S vs. BY-R	0.8511	ns	
**Fig. 2c**<br>**PAG**	Control, n = 15 mice<br>BY-S, n = 18 mice<br>BY-R, n = 15 mice	*One-way ANOVA*	*Holm-Sidak's*	Main effect of group	0.0138	*	F (2, 45) = 4.715
				Control vs. BY-S	0.0314	*	
				Control vs. BY-R	0.9995	ns	
				BY-S vs. BY-R	0.0404	*	
**Fig. 2c**<br>**DR**	Control, n = 16 mice<br>BY-S, n = 18 mice	*One-way ANOVA*	*Holm-Sidak's*	Main effect of group	1.5×10^{-5}	****	F (2, 46) = 14.25

	BY-R, n = 15 mice						
				Control vs. BY-S	0.0004	***	
				Control vs. BY-R	0.8490	ns	
				BY-S vs. BY-R	4.1×10^{-5}	****	
**Fig. 2c LC**	Control, n = 35 mice<br>BY-S, n = 30 mice<br>BY-R, n = 31 mice	*Kruskal-Wallis test*	*Dunn's*	Summary	6.2×10^{-6}	****	H = 23.98
				Control vs. BY-S	3.9×10^{-6}	****	
				Control vs. BY-R	0.0104	*	
				BY-S vs. BY-R	0.1778	ns	
**Fig. 2e**	Control, n = 22 slices<br>BY-S, n = 25 slices<br>BY-R, n = 33 slices	*Kruskal-Wallis test*	*Dunn's*	Summary	1.7×10^{-7}	****	H = 31.23
				Control vs. BY-S	0.2586	ns	
				Control vs. BY-R	2.5×10^{-7}	****	
				BY-S vs. BY-R	0.0007	***	
**Fig. 2g**	Control, n = 7 slices<br>BY-S, n = 9 slices<br>BY-R, n = 7 slices	*One-way ANOVA*	*Tukey's*	Main effect of group	0.9945	ns	F (2, 20) = 0.005533
				Control vs. BY-S	0.9995	ns	
				Control vs. BY-R	0.9998	ns	
				BY-S vs. BY-R	>0.9999	ns	
**Fig. 2i**	Control, n = 9 slices<br>BY-S, n = 9 slices<br>BY-R, n = 11 slices	*One-way ANOVA*	*Tukey's*	Main effect of group	0.0855	ns	F (2, 26) = 2.707
				Control vs. BY-S	0.0707	ns	
				Control vs. BY-R	0.3702	ns	
				BY-S vs. BY-R	0.5458	ns	

**Fig. 2k**	Control, n = 9 slices<br>BY-S, n = 8 slices<br>BY-R, n = 8 slices	*Kruskal-Wallis test*	*Dunn's*	Summary	0.0004	***	H = 15.80
				Control vs. BY-S	>0.9999	ns	
				Control vs. BY-R	0.0017	**	
				BY-S vs. BY-R	0.0015	**	
**Supplementary Fig. 5d**	Control, n = 9 slices / mice<br>BY-S, n = 8 slices / mice	*Two-sided Fisher's exact test*			0.1761	ns	
	Control, n = 9 slices / mice<br>BY-R, n = 8 slices / mice	*Two-sided Fisher's exact test*			<0.0001	****	
	BY-S, n = 8 slices / mice<br>BY-R, n = 8 slices / mice	*Two-sided Fisher's exact test*			8.1×10^{-12}	****	
**Supplementary Fig. 6b**	Control, n = 3 mice<br>BY-S, n = 3 slices<br>BY-R, n = 3 slices	*One-way ANOVA*	*Tukey's*	Main effect of group	0.0036	**	F (2, 6) = 16.66
				Control vs. BY-S	0.0057	**	
				Control vs. BY-R	0.9971	ns	
				BY-S vs. BY-R	0.0061	**	
**Supplementary Fig. 6d**	Control, n = 11 cells/ 3mice<br>BY-S, n = 11 cells/ 3mice<br>BY-R, n = 12 cells/ 3mice	*One-way ANOVA*	*Tukey's*	Main effect of group	0.0047	**	F (2, 31) = 6.403
				Control vs. BY-S	0.7750	ns	
				Control vs. BY-R	0.0056	**	
				BY-S vs. BY-R	0.0312	*	
**Fig. 3c**	Gq-saline, n = 6 slices<br>Gq-CNO, n = 4 slices	*Unpaired Two-sided t-test*			1.6×10^{-7}	****	t (8) = 23.35
**Fig. 3d**	Control, n = 13 mice<br>BY-S, n = 17 mice	*Two-way RM ANOVA*	*Tukey's*	Group × time interaction	4.5×10^{-6}	****	F (6, 102) = 6.787

	BY-R, n = 7 mice						
				Control vs. BY-S at BL	0.8990	ns	
				Control vs. BY-R at BL	0.9152	ns	
				BY-S vs. BY-R at BL	0.6928	ns	
				Control vs. BY-S at Test 1	1.4×10^{-5}	****	
				Control vs. BY-R at Test 1	0.7226	ns	
				BY-S vs. BY-R at Test 1	0.0004	***	
				Control vs. BY-S at Test 2	0.9706	ns	
				Control vs. BY-R at Test 2	0.5950	ns	
				BY-S vs. BY-R at Test 2	0.5239	ns	
				Control vs. BY-S at Test 3	2.9×10^{-5}	****	
				Control vs. BY-R atTest 3	0.5041	ns	
				BY-S vs. BY-R at Test 3	0.0002	***	
				Control BL vs. Test 1	0.9928	ns	
				Control BL vs. Test 2	0.9602	ns	
				Control BL vs. Test 3	0.9685	ns	
				Control Test 1 vs. Test 2	0.6196	ns	
				Control Test 1 vs. Test 3	>0.9999	ns	
				Control Test 2 vs. Test 3	0.6447	ns	
				BY-S BL vs. Test 1	2.7×10^{-6}	****	
				BY-S BL vs. Test 2	0.9547	ns	
				BY-S BL vs. Test 3	2.3×10^{-5}	****	
				BY-S Test 1 vs. Test 2	0.0007	***	
				BY-S Test 1 vs. Test 3	0.7365	ns	
				BY-S Test 2 vs. Test 3	0.0009	***	
				BY-R BL vs. Test 1	0.9970	ns	
				BY-R BL vs. Test 2	0.7560	ns	
				BY-R BL vs. Test 3	0.7639	ns	

				BY-R Test 1 vs. Test 2	0.8122	ns	
				BY-R Test 1 vs. Test 3	0.3557	ns	
				BY-R Test 2 vs. Test 3	0.9266	ns	
**Fig. 3e**	Test 1, n = 24 mice<br>Test 2, n = 24 mice<br>Test 3, n = 24 mice	*chi-square test*			0.0002	***	$\chi^2 = 17.46$
	Test 1, BY-S n = 17 mice<br>Test 1, BY-R n = 7 mice						
	Test 2, BY-S n = 4 mice<br>Test 2, BY-R n = 20 mice						
	Test 3, BY-S n = 16 mice<br>Test 3, BY-R n = 8 mice						
**Fig. 3e**	Test 1, n = 24 mice<br>Test 2, n = 24 mice	*Two-sided Fisher's exact test*			0.0004	***	
	Test 1, BY-S n = 17 mice<br>Test 1, BY-R n = 7 mice						
	Test 2, BY-S n = 4 mice<br>Test 2, BY-R n = 20 mice						
**Fig. 3e**	Test 1, n = 24 mice<br>Test 3, n = 24 mice	*Two-sided Fisher's exact test*			>0.9999	ns	
	Test 1, BY-S n = 17 mice<br>Test 1, BY-R n = 7 mice						
	Test 3, BY-S n = 16 mice<br>Test 3, BY-R n = 8 mice						
**Fig. 3e**	Test 2, n = 24 mice<br>Test 3, n = 24 mice	*Two-sided Fisher's exact test*			0.0010	**	
	Test 2, BY-S n = 4 mice<br>Test 2, BY-R n = 20 mice						

	Test 3, BY-S n = 16 mice<br>Test 3, BY-R n = 8 mice						
**Fig. 3g**	Control+saline, n = 10 mice<br>Control+CNO, n = 10 mice<br>BY-S+saline, n = 10 mice<br>BY-S+CNO, n = 10 mice	*Two-way RM ANOVA*	*Tukey's*	Group × time interaction	1.6×10^{-7}	****	F (9, 108) = 6.645
				Control+saline vs. Control+CNO at BL	>0.9999	ns	
				Control+saline vs. BY-S+saline at BL	0.9990	ns	
				Control+saline vs. BY-S+CNO at BL	0.9990	ns	
				Control+CNO vs. BY-S+saline at BL	0.9990	ns	
				Control+CNO vs. BY-S+CNO at BL	0.9990	ns	
				BY-S+saline vs. BY-S+CNO at BL	>0.9999	ns	
				Control+saline vs. Control+CNO at 30min	0.9710	ns	
				Control+saline vs. BY-S+saline at 30min	0.0014	**	
				Control+saline vs. BY-S+CNO at 30min	>0.9999	ns	
				Control+CNO vs. BY-S+saline at 30min	0.0002	***	
				Control+CNO vs. BY-S+CNO at 30min	0.9851	ns	
				BY-S+saline vs. BY-S+CNO at 30min	0.0034	**	
				Control+saline vs. Control+CNO at 2h	0.8953	ns	
				Control+saline vs. BY-S+saline at 2h	0.0001	***	
				Control+saline vs. BY-S+CNO at 2h	0.0758	ns	
				Control+CNO vs. BY-S+saline at 2h	1.6×10^{-6}	****	
				Control+CNO vs. BY-S+CNO at 2h	0.1264	ns	
				BY-S+saline vs. BY-S+CNO at 2h	0.1688	ns	
				Control+saline vs. Control+CNO at 6h	0.9996	ns	
				Control+saline vs. BY-S+saline at 6h	0.0006	***	
				Control+saline vs. BY-S+CNO at 6h	0.0006	***	
				Control+CNO vs. BY-S+saline at 6h	0.0003	***	

				Control+CNO vs. BY-S+CNO at 6h	0.0003	***	
				BY-S+saline vs. BY-S+CNO at 6h	0.9816	ns	
**Fig. 3h**	n = 12 mice	*Two-sided Friedman test*	*Dunn's*	Summary	0.0002	***	Q = 17.15
				BL vs. Pre	0.0005	***	
				BL vs. CNO	>0.9999	ns	
				Pre vs. CNO	0.0033	**	
**Fig. 4c**	Gi-saline, n = 6 slices<br>Gi-CNO, n = 6 slices	*Unpaired Two-sided t-test*			1.1×10^{-6}	****	t (10) = 10.44
**Fig. 4d**	Control, n = 10 mice<br>BY-S, n = 17 mice<br>BY-R, n = 9 mice	*Two-way RM ANOVA*	*Tukey's*	Group × time interaction	9.0×10^{-15}	****	F (6, 99) = 19.24
				Control vs. BY-S at BL	>0.9999	ns	
				Control vs. BY-R at BL	0.7498	ns	
				BY-S vs. BY-R at BL	0.6595	ns	
				Control vs. BY-S at Test 1	4.4×10^{-6}	****	
				Control vs. BY-R at Test 1	0.6023	ns	
				BY-S vs. BY-R at Test 1	2.1×10^{-7}	****	
				Control vs. BY-S at Test 2	0.0015	**	
				Control vs. BY-R at Test 2	0.0018	**	
				BY-S vs. BY-R at Test 2	0.8071	ns	
				Control vs. BY-S at Test 3	6.8×10^{-6}	****	
				Control vs. BY-R atTest 3	0.5510	ns	
				BY-S vs. BY-R at Test 3	0.0003	***	
				Control BL vs. Test 1	0.6015	ns	
				Control BL vs. Test 2	0.8455	ns	
				Control BL vs. Test 3	0.5258	ns	
				Control Test 1 vs. Test 2	0.9782	ns	

				Control Test 1 vs. Test 3	0.9778	ns	
				Control Test 2 vs. Test 3	0.9319	ns	
				BY-S BL vs. Test 1	3.2×10^{-7}	****	
				BY-S BL vs. Test 2	1.3×10^{-7}	****	
				BY-S BL vs. Test 3	1.5×10^{-7}	****	
				BY-S Test 1 vs. Test 2	0.6984	ns	
				BY-S Test 1 vs. Test 3	0.7986	ns	
				BY-S Test 2 vs. Test 3	0.9904	ns	
				BY-R BL vs. Test 1	0.8641	ns	
				BY-R BL vs. Test 2	0.0053	**	
				BY-R BL vs. Test 3	0.9190	ns	
				BY-R Test 1 vs. Test 2	0.0002	***	
				BY-R Test 1I vs. Test 3	0.9957	ns	
				BY-R Test 2 vs. Test 3	0.0021	**	
**Fig. 4e**	Test 1, n = 26 mice<br>Test 2, n = 26 mice<br>Test 3, n = 26 mice	*chi-square test*			0.0041	**	$\chi^2 = 10.98$
	Test 1, BY-S n = 17 mice<br>Test 1, BY-R n = 9 mice						
	Test 2, BY-S n = 26 mice<br>Test 2, BY-R n = 0 mice						
	Test 3, BY-S n = 18 mice<br>Test 3, BY-R n = 8 mice						
**Fig. 4e**	Test 1, n = 26 mice<br>Test 2, n = 26 mice	*Two-sided Fisher's exact test*			0.0017	**	
	Test 1, BY-S n = 17 mice<br>Test 1, BY-R n = 9 mice						
	Test 2, BY-S n = 26 mice						

	Test 2, BY-R n = 0 mice						
**Fig. 4e**	Test 1, n = 26 mice<br>Test 3, n = 26 mice	*Two-sided*<br>*Fisher's exact test*			>0.9999	ns	
	Test 1, BY-S n = 17 mice<br>Test 1, BY-R n = 9 mice						
	Test 3, BY-S n = 18 mice<br>Test 3, BY-R n = 8 mice						
**Fig. 4e**	Test 2, n = 26 mice<br>Test 3, n = 26 mice	*Two-sided*<br>*Fisher's exact test*			0.0042	**	
	Test 2, BY-S n = 26 mice<br>Test 2, BY-R n = 0 mice						
	Test 3, BY-S n = 18 mice<br>Test 3, BY-R n = 8 mice						
**Fig. 4g**	Control+saline, n = 9 mice<br>Control+CNO, n = 13 mice<br>BY-R+saline, n = 9 mice<br>BY-R+CNO, n = 8 mice	*Two-way RM ANOVA*	*Tukey's*	Group × time interaction	0.0005	***	F (9, 105) = 3.682
				Control+saline vs. Control+CNO at BL	0.9556	ns	
				Control+saline vs. BY-R+saline at BL	0.8416	ns	
				Control+saline vs. BY-R+CNO at BL	0.9837	ns	
				Control+CNO vs. BY-R+saline at BL	0.9710	ns	
				Control+CNO vs. BY-R+CNO at BL	0.7588	ns	
				BY-R+saline vs. BY-R+CNO at BL	0.5942	ns	
				Control+saline vs. Control+CNO at 30min	0.5518	ns	
				Control+saline vs. BY-R+saline at 30min	0.8409	ns	
				Control+saline vs. BY-R+CNO at 30min	0.0012	**	
				Control+CNO vs. BY-R+saline at 30min	0.9853	ns	
				Control+CNO vs. BY-R+CNO at 30min	6.8×10^{-5}	****	

				BY-R+saline vs. BY-R+CNO at 30min	0.0017	**	
				Control+saline vs. Control+CNO at 2h	0.9974	ns	
				Control+saline vs. BY-R+saline at 2h	0.9471	ns	
				Control+saline vs. BY-R+CNO at 2h	0.0129	*	
				Control+CNO vs. BY-R+saline at 2h	0.9648	ns	
				Control+CNO vs. BY-R+CNO at 2h	0.0015	**	
				BY-R+saline vs. BY-R+CNO at 2h	0.2435	ns	
				Control+saline vs. Control+CNO at 6h	0.9952	ns	
				Control+saline vs. BY-R+saline at 6h	0.9757	ns	
				Control+saline vs. BY-R+CNO at 6h	0.9979	ns	
				Control+CNO vs. BY-R+saline at 6h	0.9928	ns	
				Control+CNO vs. BY-R+CNO at 6h	>0.9999	ns	
				BY-R+saline vs. BY-R+CNO at 6h	0.9935	ns	
**Fig. 4i**	Control, n = 9 mice<br>BY+saline, n = 8 mice<br>BY+CNO, n = 8 mice	*Two-way RM ANOVA*	*Tukey's*	Group × time interaction	0.0022	**	$F(10, 115) = 2.988$
				Control vs. BY+saline at BL	0.9637	ns	
				Control vs. BY+CNO at BL	0.9082	ns	
				BY+saline vs. BY+CNO at BL	0.9978	ns	
				Control vs. BY+saline at 30min	0.0904	ns	
				Control vs. BY+CNO at 30min	2.2×10^{-5}	****	
				BY+saline vs. BY+CNO at 30min	0.0616	ns	
				Control vs. BY+saline at 2h	0.9708	ns	
				Control vs. BY+CNO at 2h	0.0324	*	
				BY+saline vs. BY+CNO at 2h	0.1158	ns	
				Control vs. BY+saline at 6h	>0.9999	ns	
				Control vs. BY+CNO at 6h	0.0647	ns	
				BY+saline vs. BY+CNO at 6h	0.0839	ns	

				Control vs. BY+saline at 12h	0.7856	ns	
				Control vs. BY+CNO at 12h	0.0284	*	
				BY+saline vs. BY+CNO at 12h	0.2523	ns	
				Control vs. BY+saline at 24h	0.7824	ns	
				Control vs. BY+CNO at 24h	0.9961	ns	
				BY+saline vs. BY+CNO at 24h	0.9023	ns	
**Supplementary Fig. 7a**	Control, n = 10 mice<br>BY-S, n = 14 mice<br>BY-R, n = 5 mice	*Two-way RM ANOVA*	*Tukey's*	Group × time interaction	3.0×10^{-7}	****	F (6, 78) = 8.703
				Control vs. BY-S at BL	0.9744	ns	
				Control vs. BY-R at BL	0.9993	ns	
				BY-S vs. BY-R at BL	0.9802	ns	
				Control vs. BY-S at Test 1	9.3×10^{-6}	****	
				Control vs. BY-R at Test 1	0.3638	ns	
				BY-S vs. BY-R at Test 1	0.0138	*	
				Control vs. BY-S at Test 2	7.0×10^{-5}	****	
				Control vs. BY-R at Test 2	0.4906	ns	
				BY-S vs. BY-R at Test 2	0.0254	*	
				Control vs. BY-S at Test 3	2.9×10^{-6}	****	
				Control vs. BY-R a Test 3	0.9964	ns	
				BY-S vs. BY-R at Test 3	0.0037	**	
				Control BL vs. Test 1	0.9878	ns	
				Control BL vs. Test 2	0.9823	ns	
				Control BL vs. Test 3	0.9998	ns	
				Control Test 1 vs. Test 2	0.8841	ns	
				Control Test 1 vs. Test 3	0.8811	ns	
				Control Test 2 vs. Test 3	0.9880	ns	
				BY-S BL vs. Test 1	4.4×10^{-6}	****	

				BY-S BL vs. Test 2	3.2×10^{-5}	****	
				BY-S BL vs. Test 3	3.0×10^{-6}	****	
				BY-S Test 1 vs. Test 2	0.5457	ns	
				BY-S Test 1 vs. Test 3	0.9847	ns	
				BY-S Test 2 vs. Test 3	0.4342	ns	
				BY-R BL vs. Test 1	0.6463	ns	
				BY-R BL vs. Test 2	0.6390	ns	
				BY-R BL vs. Test 3	0.9998	ns	
				BY-R Test 1 vs. Test 2	0.9558	ns	
				BY-R Test 1I vs. Test 3	0.7438	ns	
				BY-R Test 2 vs. Test 3	0.4689	ns	
**Supplementary Fig. 7b**	Test 1, n = 21 mice<br>Test 2, n = 21 mice<br>Test 3, n = 21 mice	*chi-square test*			0.9226	ns	$\chi^2 = 0.1611$
	Test 1, BY-S n = 15 mice<br>Test 1, BY-R n = 6 mice						
	Test 2, BY-S n = 16 mice<br>Test 2, BY-R n = 5 mice						
	Test 3, BY-S n = 15 mice<br>Test 3, BY-R n = 6 mice						
**Supplementary Fig. 7c**	Control+saline, n = 11 mice<br>Control+CNO, n = 10 mice<br>BY-S+saline, n = 14 mice<br>BY-S+CNO, n = 13 mice	*Two-way RM ANOVA*	*Tukey's*	Group × time interaction	5.5×10^{-8}	****	F (9, 132) = 6.797
				Control+saline vs. Control+CNO at BL	0.9998	ns	
				Control+saline vs. BY-S+saline at BL	0.7319	ns	
				Control+saline vs. BY-S+CNO at BL	0.9067	ns	
				Control+CNO vs. BY-S+saline at BL	0.8156	ns	

				Control+CNO vs. BY-S+CNO at BL	0.9457	ns	
				BY-S+saline vs. BY-S+CNO at BL	0.9871	ns	
				Control+saline vs. Control+CNO at 30min	0.1333	ns	
				Control+saline vs. BY-S+saline at 30min	6.1×10^{-6}	****	
				Control+saline vs. BY-S+CNO at 30min	0.2484	ns	
				Control+CNO vs. BY-S+saline at 30min	<0.0001	***	
				Control+CNO vs. BY-S+CNO at 30min	0.0104	*	
				BY-S+saline vs. BY-S+CNO at 30min	0.0019	**	
				Control+saline vs. Control+CNO at 2h	0.6355	ns	
				Control+saline vs. BY-S+saline at 2h	1.2×10^{-6}	****	
				Control+saline vs. BY-S+CNO at 2h	0.0074	**	
				Control+CNO vs. BY-S+saline at 2h	0.0003	***	
				Control+CNO vs. BY-S+CNO at 2h	0.0121	*	
				BY-S+saline vs. BY-S+CNO at 2h	0.0047	**	
				Control+saline vs. Control+CNO at 6h	0.6660	ns	
				Control+saline vs. BY-S+saline at 6h	1.2×10^{-5}	****	
				Control+saline vs. BY-S+CNO at 6h	3.3×10^{-5}	****	
				Control+CNO vs. BY-S+saline at 6h	0.0007	***	
				Control+CNO vs. BY-S+CNO at 6h	0.0014	**	
				BY-S+saline vs. BY-S+CNO at 6h	0.1871	ns	
**Supplementary Fig. 7d**	n = 9 mice	*Two-sided Friedman test*	*Dunn's*	Summary	5.5×10^{-5}	****	Q = 14.80
				BL vs. Pre	0.0201	*	
				BL vs. CNO	>0.9999	ns	
				Pre vs. CNO	0.0008	***	
**Supplementary Fig. 7f**	Gq-saline, n = 8 slices<br>Gq-CNO, n = 9 slices	*Unpaired Two-sided t-test*			<0.0001	****	t (15) = 39.30
**Supplementary**	Control, n = 10 mice	*Two-way RM ANOVA*	*Tukey's*	Group × time interaction	$1,7 \times 10^{-6}$	****	F (6, 78) = 7.693

**Fig. 8a**	BY-S, n = 13 mice<br>BY-R, n = 6 mice						
				Control vs. BY-S at BL	0.9679	ns	
				Control vs. BY-R at BL	0.9795	ns	
				BY-S vs. BY-R at BL	0.9963	ns	
				Control vs. BY-S at Test 1	4.5×10^{-7}	****	
				Control vs. BY-R at Test 1	0.9780	ns	
				BY-S vs. BY-R at Test 1	0.0002	***	
				Control vs. BY-S at Test 2	4.1×10^{-5}	****	
				Control vs. BY-R at Test 2	0.9786	ns	
				BY-S vs. BY-R at Test 2	0.0047	**	
				Control vs. BY-S at Test 3	0.0006	***	
				Control vs. BY-R at Test 3	0.9899	ns	
				BY-S vs. BY-R at Test 3	0.0078	**	
				Control BL vs. Test 1	0.9999	ns	
				Control BL vs. Test 2	0.9997	ns	
				Control BL vs. Test 3	>0.9999	ns	
				Control Test 1 vs. Test 2	0.9965	ns	
				Control Test 1 vs. Test 3	>0.9999	ns	
				Control Test 2 vs. Test 3	0.9996	ns	
				BY-S BL vs. Test 1	8.7×10^{-6}	****	
				BY-S BL vs. Test 2	1.1×10^{-5}	****	
				BY-S BL vs. Test 3	2.4×10^{-6}	****	
				BY-S Test 1 vs. Test 2	0.9993	ns	
				BY-S Test 1 vs. Test 3	0.9979	ns	
				BY-S Test 2 vs. Test 3	0.9927	ns	
				BY-R BL vs. Test 1	0.9996	ns	
				BY-R BL vs. Test 2	0.9666	ns	

				BY-R BL vs. Test 3	>0.9999	ns	
				BY-R Test 1 vs. Test 2	0.9370	ns	
				BY-R Test 1I vs. Test 3	>0.9999	ns	
				BY-R Test 2 vs. Test 3	0.9736	ns	
**Supplementary Fig. 8b**	Test 1, n = 19 mice<br>Test 2, n = 19 mice<br>Test 3, n = 19 mice	*chi-square test*			0.9168	ns	$\chi^2 = 0.1738$
	Test 1, BY-S n = 13 mice<br>Test 1, BY-R n = 6 mice						
	Test 2, BY-S n = 14 mice<br>Test 2, BY-R n = 5 mice						
	Test 3, BY-S n = 14 mice<br>Test 3, BY-R n = 5 mice						
**Supplementary Fig. 8c**	Control+saline, n = 10 mice<br>Control+CNO, n = 10 mice<br>BY-R+saline, n = 5 mice<br>BY-R+CNO, n = 5 mice	*Two-way RM ANOVA*	*Tukey's*	Group × time interaction	0.9179	ns	F (9, 78) = 0.4251
				Control+saline vs. Control+CNO at BL	0.6870	ns	
				Control+saline vs. BY-R+saline at BL	0.9971	ns	
				Control+saline vs. BY-R+CNO at BL	0.9971	ns	
				Control+CNO vs. BY-R+saline at BL	0.6311	ns	
				Control+CNO vs. BY-R+CNO at BL	0.6311	ns	
				BY-R+saline vs. BY-R+CNO at BL	>0.9999	ns	
				Control+saline vs. Control+CNO at 30min	0.9749	ns	
				Control+saline vs. BY-R+saline at 30min	0.9883	ns	
				Control+saline vs. BY-R+CNO at 30min	0.8790	ns	
				Control+CNO vs. BY-R+saline at 30min	0.9052	ns	
				Control+CNO vs. BY-R+CNO at 30min	0.9642	ns	

				BY-R+saline vs. BY-R+CNO at 30min	0.8007	ns	
				Control+saline vs. Control+CNO at 2h	0.8704	ns	
				Control+saline vs. BY-R+saline at 2h	0.9473	ns	
				Control+saline vs. BY-R+CNO at 2h	0.2651	ns	
				Control+CNO vs. BY-R+saline at 2h	>0.9999	ns	
				Control+CNO vs. BY-R+CNO at 2h	0.9469	ns	
				BY-R+saline vs. BY-R+CNO at 2h	0.9800	ns	
				Control+saline vs. Control+CNO at 6h	>0.9999	ns	
				Control+saline vs. BY-R+saline at 6h	0.4300	ns	
				Control+saline vs. BY-R+CNO at 6h	0.9950	ns	
				Control+CNO vs. BY-R+saline at 6h	0.5519	ns	
				Control+CNO vs. BY-R+CNO at 6h	0.9957	ns	
				BY-R+saline vs. BY-R+CNO at 6h	0.8603	ns	
**Supplementary Fig. 8d**	Control, n = 10 mice<br>BY+saline, n = 8 mice<br>BY+CNO, n = 9 mice	*Two-way RM ANOVA*	*Tukey's*	Group × time interaction	0.2649	ns	F (2, 24) = 1.405
				Control vs. BY+saline at BL	0.8308	ns	
				Control vs. BY+CNO at BL	0.9761	ns	
				BY+saline vs. BY+CNO at BL	0.9710	ns	
				Control vs. BY+saline at BY-15min	0.8515	ns	
				Control vs. BY+CNO at BY-15min	0.2933	ns	
				BY+saline vs. BY+CNO at BY-15min	0.0841	ns	
**Supplementary Fig. 8f**	Gi-saline, n = 8 slices<br>Gi-CNO, n = 7 slices	*Unpaired Two-sided t-test*			2.0×10^{-5}	****	t (13) = 6.517
**Fig. 6b**	Control+mCherry, n = 20 mice<br>Control+Gq, n = 10 mice<br>Control+Gi, n = 7 mice<br>BY+mCherry, n = 20 mice	*Two-way RM ANOVA*	*Tukey's*	Group × time interaction	5.6×10^{-13}	****	F (15, 261) = 7.088

	BY+Gq, n = 20 mice<br>BY+Gi, n = 16 mice						
				Control+mCherry vs. Control+Gq at BL	0.3401	ns	
				Control+mCherry vs. Control+Gi at BL	>0.9999	ns	
				Control+mCherry vs. BY+mCherry at BL	0.8616	ns	
				Control+mCherry vs. BY+Gq at BL	0.5857	ns	
				Control+mCherry vs. BY+Gi at BL	0.9988	ns	
				Control+Gq vs. Control+Gi at BL	0.7242	ns	
				Control+Gq vs. BY+mCherry at BL	0.9172	ns	
				Control+Gq vs. BY+Gq at BL	0.9666	ns	
				Control+Gq vs. BY+Gi at BL	0.2466	ns	
				Control+Gi vs. BY+mCherry at BL	0.9772	ns	
				Control+Gi vs. BY+Gq at BL	0.9265	ns	
				Control+Gi vs. BY+Gi at BL	>0.9999	ns	
				BY+mCherry vs. BY+Gq at BL	0.9996	ns	
				BY+mCherry vs. BY+Gi at BL	0.7220	ns	
				BY+Gq vs. BY+Gi at BL	0.4210	ns	
				Control+mCherry vs. Control+Gq at 30min	0.9999	ns	
				Control+mCherry vs. Control+Gi at 30min	>0.9999	ns	
				Control+mCherry vs. BY+mCherry at 30min	4.3×10^{-5}	****	
				Control+mCherry vs. BY+Gq at 30min	0.9266	ns	
				Control+mCherry vs. BY+Gi at 30min	2.1×10^{-10}	****	
				Control+Gq vs. Control+Gi at 30min	>0.9999	ns	
				Control+Gq vs. BY+mCherry at 30min	0.0012	**	
				Control+Gq vs. BY+Gq at 30min	0.9867	ns	
				Control+Gq vs. BY+Gi at 30min	0.0001	***	
				Control+Gi vs. BY+mCherry at 30min	0.0012	**	
				Control+Gi vs. BY+Gq at 30min	0.9659	ns	

				Control+Gi vs. BY+Gi at 30min	0.0004	***	
				BY+mCherry vs. BY+Gq at 30min	0.0044	**	
				BY+mCherry vs. BY+Gi at 30min	0.4665	ns	
				BY+Gq vs. BY+Gi at 30min	6.8×10^{-6}	****	
				Control+mCherry vs. Control+Gq at 2h	>0.9999	ns	
				Control+mCherry vs. Control+Gi at 2h	0.9790	ns	
				Control+mCherry vs. BY+mCherry at 2h	0.0015	**	
				Control+mCherry vs. BY+Gq at 2h	>0.9999	ns	
				Control+mCherry vs. BY+Gi at 2h	3.0×10^{-12}	****	
				Control+Gq vs. Control+Gi at 2h	0.9997	ns	
				Control+Gq vs. BY+mCherry at 2h	0.1105	ns	
				Control+Gq vs. BY+Gq at 2h	>0.9999	ns	
				Control+Gq vs. BY+Gi at 2h	0.0064	**	
				Control+Gi vs. BY+mCherry at 2h	0.0184	*	
				Control+Gi vs. BY+Gq at 2h	0.9846	ns	
				Control+Gi vs. BY+Gi at 2h	0.0003	***	
				BY+mCherry vs. BY+Gq at 2h	0.0110	*	
				BY+mCherry vs. BY+Gi at 2h	0.2358	ns	
				BY+Gq vs. BY+Gi at 2h	9.9×10^{-6}	****	
				Control+mCherry vs. Control+Gq at 6h	0.9926	ns	
				Control+mCherry vs. Control+Gi at 6h	0.9941	ns	
				Control+mCherry vs. BY+mCherry at 6h	0.0002	***	
				Control+mCherry vs. BY+Gq at 6h	>0.9999	ns	
				Control+mCherry vs. BY+Gi at 6h	2.0×10^{-12}	****	
				Control+Gq vs. Control+Gi at 6h	>0.9999	ns	
				Control+Gq vs. BY+mCherry at 6h	0.0412	*	
				Control+Gq vs. BY+Gq at 6h	0.9922	ns	
				Control+Gq vs. BY+Gi at 6h	0.0041	**	

				Control+Gi vs. BY+mCherry at 6h	0.0153	*	
				Control+Gi vs. BY+Gq at 6h	0.9947	ns	
				Control+Gi vs. BY+Gi at 6h	0.0027	**	
				BY+mCherry vs. BY+Gq at 6h	0.0115	*	
				BY+mCherry vs. BY+Gi at 6h	0.1384	ns	
				BY+Gq vs. BY+Gi at 6h	2.2×10^{-5}	****	
**Fig. 6c**	Saline, n = 20 mice<br>Gq, n = 20 mice<br>Gi, n = 20 mice	*chi-square test*			3.5×10^{-5}	****	$\chi^2 = 20.48$
	Saline, BY-S n = 15 mice<br>Saline, BY-R n = 5 mice						
	Gq, BY-S n = 7 mice<br>Gq, BY-R n = 13 mice						
	Gi, BY-S n = 20 mice<br>Gi, BY-R n = 0 mice						
**Fig. 6c**	Saline, n = 20 mice<br>Gq, n = 20 mice	*Two-sided*<br>*Fisher's exact test*			0.0248	*	
	Saline, BY-S n = 15 mice<br>Saline, BY-R n = 5 mice						
	Gq, BY-S n = 7 mice<br>Gq, BY-R n = 13 mice						
**Fig. 6c**	Saline, n = 20 mice<br>Gi, n = 20 mice	*Two-sided*<br>*Fisher's exact test*			0.0471	*	
	Saline, BY-S n = 15 mice<br>Saline, BY-R n = 5 mice						
	Gi, BY-S n = 20 mice<br>Gi, BY-R n = 0 mice						
**Fig. 6c**	Gq, n = 20 mice	*Two-sided*			1.3×10^{-5}	****	

	Gi, n = 20 mice	*Fisher's exact test*					
	Gq, BY-S n = 7 mice<br>Gq, BY-R n = 13 mice						
	Gi, BY-S n = 20 mice<br>Gi, BY-R n = 0 mice						
**Fig. 6d**	Control+mCherry, n = 10 mice<br>Control+Gq, n = 9 mice<br>Control+Gi, n = 9 mice<br>BY+mCherry, n = 20 mice<br>BY+Gq, n = 9 mice<br>BY+Gi, n = 10 mice	*Two-way RM ANOVA*	*Tukey's*	Group × time interaction	3.6×10^{-6}	****	F (15, 183) = 3.955
				Control+mCherry vs. Control+Gq at BL	0.9987	ns	
				Control+mCherry vs. Control+Gi at BL	0.9702	ns	
				Control+mCherry vs. BY+mCherry at BL	0.9976	ns	
				Control+mCherry vs. BY+Gq at BL	0.9987	ns	
				Control+mCherry vs. BY+Gi at BL	0.9768	ns	
				Control+Gq vs. Control+Gi at BL	0.9986	ns	
				Control+Gq vs. BY+mCherry at BL	0.9288	ns	
				Control+Gq vs. BY+Gq at BL	>0.9999	ns	
				Control+Gq vs. BY+Gi at BL	0.9994	ns	
				Control+Gi vs. BY+mCherry at BL	0.7159	ns	
				Control+Gi vs. BY+Gq at BL	0.9986	ns	
				Control+Gi vs. BY+Gi at BL	>0.9999	ns	
				BY+mCherry vs. BY+Gq at BL	0.9288	ns	
				BY+mCherry vs. BY+Gi at BL	0.7107	ns	
				BY+Gq vs. BY+Gi at BL	0.9994	ns	
				Control+mCherry vs. Control+Gq at 30min	0.9976	ns	
				Control+mCherry vs. Control+Gi at 30min	0.9873	ns	

				Control+mCherry vs. BY+mCherry at 30min	0.0196	*	
				Control+mCherry vs. BY+Gq at 30min	0.5598	ns	
				Control+mCherry vs. BY+Gi at 30min	0.0446	*	
				Control+Gq vs. Control+Gi at 30min	>0.9999	ns	
				Control+Gq vs. BY+mCherry at 30min	0.0099	**	
				Control+Gq vs. BY+Gq at 30min	0.4220	ns	
				Control+Gq vs. BY+Gi at 30min	0.0248	*	
				Control+Gi vs. BY+mCherry at 30min	0.0056	**	
				Control+Gi vs. BY+Gq at 30min	0.3621	ns	
				Control+Gi vs. BY+Gi at 30min	0.0170	*	
				BY+mCherry vs. BY+Gq at 30min	0.9895	ns	
				BY+mCherry vs. BY+Gi at 30min	>0.9999	ns	
				BY+Gq vs. BY+Gi at 30min	0.9855	ns	
				Control+mCherry vs. Control+Gq at 2h	0.9785	ns	
				Control+mCherry vs. Control+Gi at 2h	0.9783	ns	
				Control+mCherry vs. BY+mCherry at 2h	0.0069	**	
				Control+mCherry vs. BY+Gq at 2h	0.2245	ns	
				Control+mCherry vs. BY+Gi at 2h	0.0099	**	
				Control+Gq vs. Control+Gi at 2h	>0.9999	ns	
				Control+Gq vs. BY+mCherry at 2h	0.0520	ns	
				Control+Gq vs. BY+Gq at 2h	0.4719	ns	
				Control+Gq vs. BY+Gi at 2h	0.0479	*	
				Control+Gi vs. BY+mCherry at 2h	0.0207	*	
				Control+Gi vs. BY+Gq at 2h	0.4038	ns	
				Control+Gi vs. BY+Gi at 2h	0.0266	*	
				BY+mCherry vs. BY+Gq at 2h	0.9976	ns	
				BY+mCherry vs. BY+Gi at 2h	0.9991	ns	
				BY+Gq vs. BY+Gi at 2h	0.9805	ns	

				Control+mCherry vs. Control+Gq at 6h	0.9989	ns	
				Control+mCherry vs. Control+Gi at 6h	0.9996	ns	
				Control+mCherry vs. BY+mCherry at 6h	0.0831	ns	
				Control+mCherry vs. BY+Gq at 6h	0.3524	ns	
				Control+mCherry vs. BY+Gi at 6h	0.0624	ns	
				Control+Gq vs. Control+Gi at 6h	0.9746	ns	
				Control+Gq vs. BY+mCherry at 6h	0.0407	*	
				Control+Gq vs. BY+Gq at 6h	0.2173	ns	
				Control+Gq vs. BY+Gi at 6h	0.0315	*	
				Control+Gi vs. BY+mCherry at 6h	0.0479	*	
				Control+Gi vs. BY+Gq at 6h	0.3767	ns	
				Control+Gi vs. BY+Gi at 6h	0.0416	*	
				BY+mCherry vs. BY+Gq at 6h	0.9997	ns	
				BY+mCherry vs. BY+Gi at 6h	0.9994	ns	
				BY+Gq vs. BY+Gi at 6h	0.9944	ns	
**Fig. 6e**	Saline, n = 20 mice<br>Gq, n = 10 mice<br>Gi, n = 10 mice	*chi-square test*			>0.9999	ns	
	Saline, BY-S n = 14 mice<br>Saline, BY-R n = 6 mice						
	Gq, BY-S n = 7 mice<br>Gq, BY-R n = 3 mice						
	Gi, BY-S n = 7 mice<br>Gi, BY-R n = 3 mice						
**Fig. 6e**	Saline, n = 20 mice<br>Gq, n = 10 mice	*Two-sided*<br>*Fisher's exact test*			>0.9999	ns	
	Saline, BY-S n = 14 mice<br>Saline, BY-R n = 6 mice						

	Gq, BY-S n = 7 mice<br>Gq, BY-R n = 3 mice						
**Fig. 6e**	Saline, n = 20 mice<br>Gi, n = 10 mice	*Two-sided*<br>*Fisher's exact test*			>0.9999	ns	
	Saline, BY-S n = 14 mice<br>Saline, BY-R n = 6 mice						
	Gi, BY-S n = 7 mice<br>Gi, BY-R n = 3 mice						
**Fig. 6g**	Control+mCherry, n = 20 mice<br>Control+Gq, n = 10 mice<br>BY-S+mCherry, n = 16 mice<br>BY-S+Gq, n = 15 mice	*Two-way RM ANOVA*	*Tukey's*	Group × time interaction	2.0×10^{-15}	****	F (9, 171) = 12.70
				Control+mCherry vs. Control+Gq at BL	0.1994	ns	
				Control+mCherry vs. BY-S+mCherry at BL	0.5793	ns	
				Control+mCherry vs. BY-S+Gq at BL	0.6260	ns	
				Control+Gq vs. BY-S+mCherry at BL	0.8567	ns	
				Control+Gq vs. BY-S+Gq at BL	0.7415	ns	
				BY-S+mCherry vs. BY-S+Gq at BL	0.9977	ns	
				Control+mCherry vs. Control+Gq at 30min	0.9955	ns	
				Control+mCherry vs. BY-S+mCherry at 30min	7.9×10^{-11}	****	
				Control+mCherry vs. BY-S+Gq at 30min	0.7642	ns	
				Control+Gq vs. BY-S+mCherry at 30min	4.1×10^{-5}	****	
				Control+Gq vs. BY-S+Gq at 30min	0.8787	ns	
				BY-S+mCherry vs. BY-S+Gq at 30min	0.0013	**	
				Control+mCherry vs. Control+Gq at 2h	0.9996	ns	
				Control+mCherry vs. BY-S+mCherry at 2h	5.2×10^{-12}	****	
				Control+mCherry vs. BY-S+Gq at 2h	0.0091	**	
				Control+Gq vs. BY-S+mCherry at 2h	0.0051	**	

				Control+Gq vs. BY-S+Gq at 2h	0.1118	ns	
				BY-S+mCherry vs. BY-S+Gq at 2h	0.2711	ns	
				Control+mCherry vs. Control+Gq at 6h	0.9453	ns	
				Control+mCherry vs. BY-S+mCherry at 6h	5.5×10^{-12}	****	
				Control+mCherry vs. BY-S+Gq at 6h	5.5×10^{-11}	****	
				Control+Gq vs. BY-S+mCherry at 6h	0.0034	**	
				Control+Gq vs. BY-S+Gq at 6h	0.0036	**	
				BY-S+mCherry vs. BY-S+Gq at 6h	0.9968	ns	
**Fig. 6h**	Control+mCherry, n = 20 mice<br>Control+Gi, n = 7 mice<br>BY-R+mCherry, n = 4 mice<br>BY-R+Gi, n = 5 mice	*Two-way RM ANOVA*	*Tukey's*	Group × time interaction	0.7099	ns	$F(9, 96) = 0.6972$
				Control+mCherry vs. Control+Gi at BL	>0.9999	ns	
				Control+mCherry vs. BY-R+mCherry at BL	0.9994	ns	
				Control+mCherry vs. BY-R+Gi at BL	0.9944	ns	
				Control+Gi vs. BY-R+mCherry at BL	0.9995	ns	
				Control+Gi vs. BY-R+Gi at BL	0.9986	ns	
				BY-R+mCherry vs. BY-R+Gi at BL	>0.9999	ns	
				Control+mCherry vs. Control+Gi at 30min	0.9998	ns	
				Control+mCherry vs. BY-R+mCherry at 30min	0.9429	ns	
				Control+mCherry vs. BY-R+Gi at 30min	0.4799	ns	
				Control+Gi vs. BY-R+mCherry at 30min	0.9367	ns	
				Control+Gi vs. BY-R+Gi at 30min	0.5751	ns	
				BY-R+mCherry vs. BY-R+Gi at 30min	0.5564	ns	
				Control+mCherry vs. Control+Gi at 2h	0.8969	ns	
				Control+mCherry vs. BY-R+mCherry at 2h	0.7989	ns	
				Control+mCherry vs. BY-R+Gi at 2h	0.8917	ns	
				Control+Gi vs. BY-R+mCherry at 2h	0.6983	ns	

				Control+Gi vs. BY-R+Gi at 2h	0.9825	ns	
				BY-R+mCherry vs. BY-R+Gi at 2h	0.6589	ns	
				Control+mCherry vs. Control+Gi at 6h	0.9537	ns	
				Control+mCherry vs. BY-R+mCherry at 6h	0.7247	ns	
				Control+mCherry vs. BY-R+Gi at 6h	0.9440	ns	
				Control+Gi vs. BY-R+mCherry at 6h	0.9375	ns	
				Control+Gi vs. BY-R+Gi at 6h	0.8411	ns	
				BY-R+mCherry vs. BY-R+Gi at 6h	0.6062	ns	
**Fig. 6i**	Control+mCherry, n = 10 mice<br>Control+Gq, n = 9 mice<br>BY-S+mCherry, n = 13 mice<br>BY-S+Gq, n = 6 mice	*Two-way RM ANOVA*	*Tukey's*	Group × time interaction	6.0×10^{-14}	****	$F(9, 102) = 13.41$
				Control+mCherry vs. Control+Gq at BL	0.9798	ns	
				Control+mCherry vs. BY-S+mCherry at BL	0.9968	ns	
				Control+mCherry vs. BY-S+Gq at BL	0.3428	ns	
				Control+Gq vs. BY-S+mCherry at BL	0.8977	ns	
				Control+Gq vs. BY-S+Gq at BL	0.5584	ns	
				BY-S+mCherry vs. BY-S+Gq at BL	0.0647	ns	
				Control+mCherry vs. Control+Gq at 30min	0.9712	ns	
				Control+mCherry vs. BY-S+mCherry at 30min	2.0×10^{-5}	****	
				Control+mCherry vs. BY-S+Gq at 30min	0.5828	ns	
				Control+Gq vs. BY-S+mCherry at 30min	4.1×10^{-5}	****	
				Control+Gq vs. BY-S+Gq at 30min	0.7401	ns	
				BY-S+mCherry vs. BY-S+Gq at 30min	0.0077	**	
				Control+mCherry vs. Control+Gq at 2h	0.8931	ns	
				Control+mCherry vs. BY-S+mCherry at 2h	5.1×10^{-5}	****	
				Control+mCherry vs. BY-S+Gq at 2h	0.0107	*	
				Control+Gq vs. BY-S+mCherry at 2h	0.0004	***	

				Control+Gq vs. BY-S+Gq at 2h	0.0377	*	
				BY-S+mCherry vs. BY-S+Gq at 2h	0.3454	ns	
				Control+mCherry vs. Control+Gq at 6h	0.9816	ns	
				Control+mCherry vs. BY-S+mCherry at 6h	0.0012	**	
				Control+mCherry vs. BY-S+Gq at 6h	0.0187	*	
				Control+Gq vs. BY-S+mCherry at 6h	0.0010	**	
				Control+Gq vs. BY-S+Gq at 6h	0.0098	**	
				BY-S+mCherry vs. BY-S+Gq at 6h	0.2355	ns	
**Fig. 6j**	Control+mCherry, n = 10 mice<br>Control+Gi, n = 9 mice<br>BYR+mCherry, n = 7 mice<br>BY-R+Gi, n = 3 mice	*Two-way RM ANOVA*	*Tukey's*	Group × time interaction	0.3280	ns	F (9, 75) = 1.168
				Control+mCherry vs. Control+Gi at BL	0.8698	ns	
				Control+mCherry vs. BY-R+mCherry at BL	0.9220	ns	
				Control+mCherry vs. BY-R+Gi at BL	0.7184	ns	
				Control+Gi vs. BY-R+mCherry at BL	0.5620	ns	
				Control+Gi vs. BY-R+Gi at BL	0.9945	ns	
				BY-R+mCherry vs. BY-R+Gi at BL	0.4154	ns	
				Control+mCherry vs. Control+Gi at 30min	0.9218	ns	
				Control+mCherry vs. BY-R+mCherry at 30min	0.8939	ns	
				Control+mCherry vs. BY-R+Gi at 30min	0.0016	**	
				Control+Gi vs. BY-R+mCherry at 30min	0.9994	ns	
				Control+Gi vs. BY-R+Gi at 30min	0.0008	***	
				BY-R+mCherry vs. BY-R+Gi at 30min	0.0019	**	
				Control+mCherry vs. Control+Gi at 2h	0.8923	ns	
				Control+mCherry vs. BY-R+mCherry at 2h	0.9939	ns	
				Control+mCherry vs. BY-R+Gi at 2h	0.6006	ns	
				Control+Gi vs. BY-R+mCherry at 2h	0.9931	ns	

				Control+Gi vs. BY-R+Gi at 2h	0.7388	ns	
				BY-R+mCherry vs. BY-R+Gi at 2h	0.7039	ns	
				Control+mCherry vs. Control+Gi at 6h	0.9897	ns	
				Control+mCherry vs. BY-R+mCherry at 6h	0.9971	ns	
				Control+mCherry vs. BY-R+Gi at 6h	>0.9999	ns	
				Control+Gi vs. BY-R+mCherry at 6h	0.9468	ns	
				Control+Gi vs. BY-R+Gi at 6h	0.9915	ns	
				BY-R+mCherry vs. BY-R+Gi at 6h	0.9943	ns	
**Supplementary Fig. 10c**	Gq-saline, n = 12 slices<br>Gq-CNO, n = 11 slices	*Mann-Whitney test*			1.5×10^{-6}	****	U = 0
**Supplementary Fig. 10e**	Gi-saline, n = 8 slices<br>Gi-CNO, n = 9 slices	*Unpaired Two-sided t-test*			2.7×10^{-5}	****	t (15) = 5.935
**Supplementary Fig. 11c**	Gq-saline, n = 10 slices<br>Gq-CNO, n = 9 slices	*Unpaired Two-sided t-test*			6.1×10^{-13}	****	t (17) = 19.15
**Supplementary Fig. 11e**	Gi-saline, n = 10 slices<br>Gi-CNO, n = 9 slices	*Unpaired Two-sided t-test*			0.0023	**	t (17) = 3.580
**Supplementary Fig. 12b**	Control, n = 10 mice<br>BY+mCherry, n = 8 mice<br>BY+Gi, n = 8 mice	*Two-way RM ANOVA*	*Tukey's*	Group × time interaction	0.0333	*	F (10, 115) = 2.060
				Control vs. BY+mCherry at BL	0.9177	ns	
				Control vs. BY+Gi at BL	0.8625	ns	
				BY+mCherryl vs. BY+Gi at BL	0.5702	ns	
				Control vs. BY+mCherry at 30min	0.1213	ns	
				Control vs. BY+Gi at 30min	0.0034	**	
				BY+mCherryl vs. BY+Gi at 30min	0.4342	ns	
				Control vs. BY+mCherry at 2h	0.9323	ns	
				Control vs. BY+Gi at 2h	0.0981	ns	
				BY+mCherryl vs. BY+Gi at 2h	0.3150	ns	

				Control vs. BY+mCherry at 6h	>0.9999	ns	
				Control vs. BY+Gi at 6h	0.1897	ns	
				BY+mCherryl vs. BY+Gi at 6h	0.1967	ns	
				Control vs. BY+mCherry at 12h	0.7113	ns	
				Control vs. BY+Gi at 12h	0.2391	ns	
				BY+mCherryl vs. BY+Gi at 12h	0.8610	ns	
				Control vs. BY+mCherry at 24h	0.6217	ns	
				Control vs. BY+Gi at 24h	0.9820	ns	
				BY+mCherry vs. BY+Gi at 24h	0.6524	ns	
**Supplementary Fig. 12c**	Control, n = 10 mice<br>BY+mCherry, n = 8 mice<br>BY+Gi, n = 8 mice	*Two-way RM ANOVA*	*Tukey's*	Group × time interaction	0.3361	ns	F (10, 125) = 1.143
				Control vs. BY+mCherry at BL	0.9177	ns	
				Control vs. BY+Gi at BL	0.9650	ns	
				BY+mCherry vs. BY+Gi at BL	0.9799	ns	
				Control vs. BY+mCherry at 30min	0.1213	ns	
				Control vs. BY+Gi at 30min	0.2915	ns	
				BY+mCherry vs. BY+Gi at 30min	0.7966	ns	
				Control vs. BY+mCherry at 2h	0.9323	ns	
				Control vs. BY+Gi at 2h	0.5717	ns	
				BY+mCherry vs. BY+Gi at 2h	0.8832	ns	
				Control vs. BY+mCherry at 6h	>0.9999	ns	
				Control vs. BY+Gi at 6h	0.0835	ns	
				BY+mCherry vs. BY+Gi at 6h	0.0924	ns	
				Control vs. BY+mCherry at 12h	0.7113	ns	
				Control vs. BY+Gi at 12h	0.4584	ns	
				BY+mCherry vs. BY+Gi at 12h	0.9771	ns	
				Control vs. BY+mCherry at 24h	0.6217	ns	

				Control vs. BY+Gi at 24h	0.8138	ns	
				BY+mCherry vs. BY+Gi at 24h	0.9595	ns	
**Supplementary Fig. 13a**	n = 15 mice	*Two-sided Friedman test*	*Dunn's*	Summary	6.0 x 10^{-6}	****	Q = 24.03
				BL vs. Pre	3.9 x 10^{-6}	****	
				BL vs. CNO	0.0140	*	
				Pre vs. CNO	0.1338	ns	
**Supplementary Fig. 13b**	n = 9 mice	*Two-sided Friedman test*	*Dunn's*	Summary	7.1 x 10^{-6}	****	Q = 14.80
				BLvs. Pre	0.0008	***	
				BL vs. CNO	>0.9999	ns	
				Pre vs. CNO	0.0201	*	
**Supplementary Fig. 15b**	ACSF, n = 5 cells/4 mice<br>ACSF+D1/D2 antagonist, n = 5 cells/ 4 mice<br>ACSF+NBQX, n = 5 cells/4 mice	*One-way ANOVA*	*Tukey's*	Main effect of group	0.0052	**	F (2, 12) =8.395
				ACSF vs. ACSF+D1/D2 antagonist	>0.9999	ns	
				ACSF vs. ACSF+NBQX	0.0111	*	
				ACSF+D1/D2 antagonist vs. ACSF+NBQX	0.0131	*	
**Supplementary Fig. 15c**	ACSF, n = 5 cells/4 mice<br>ACSF+D1/D2 antagonist, n = 5 cells/ 4 mice<br>ACSF+NBQX, n = 5 cells/4 mice	*One-way ANOVA*	*Tukey's*	Main effect of group	0.0002	***	F (2, 12) =19.73
				ACSF vs. ACSF+D1/D2 antagonist	>0.9999	ns	
				ACSF vs. ACSF+NBQX	0.0134	*	
				ACSF+D1/D2 antagonist vs. ACSF+NBQX	0.0075	**	
