# Supplementary material for: Midbrain glutamatergic circuit mechanism of resilience to socially transferred allodynia in male mice
Source: Nat Commun. 2024 Jun 10;15:4947. doi: 10.1038/s41467-024-49340-8 (PMC11164890; doi:10.1038/s41467-024-49340-8)
Supplement: Supplementary file 3 — Reporting Summary [file 41467_2024_49340_MOESM3_ESM.pdf]

nature portfolio

Corresponding author(s): Ming-Hu Han, Jun-Li Cao, Hongxing Zhang

Last updated by author(s): Apr 28, 2024

Reporting Summary

Nature Portfolio wishes to improve the reproducibility of the work that we publish. This form provides structure for consistency and transparency in reporting. For further information on Nature Portfolio policies, see our Editorial Policies and the Editorial Policy Checklist.

Statistics

For all statistical analyses, confirm that the following items are present in the figure legend, table legend, main text, or Methods section.

n/a	Confirmed	
☐	☒	The exact sample size (*n*) for each experimental group/condition, given as a discrete number and unit of measurement
☐	☒	A statement on whether measurements were taken from distinct samples or whether the same sample was measured repeatedly
☐	☒	The statistical test(s) used AND whether they are one- or two-sided<br>*Only common tests should be described solely by name; describe more complex techniques in the Methods section.*
☐	☒	A description of all covariates tested
☐	☒	A description of any assumptions or corrections, such as tests of normality and adjustment for multiple comparisons
☐	☒	A full description of the statistical parameters including central tendency (e.g. means) or other basic estimates (e.g. regression coefficient) AND variation (e.g. standard deviation) or associated estimates of uncertainty (e.g. confidence intervals)
☐	☒	For null hypothesis testing, the test statistic (e.g. *F*, *t*, *r*) with confidence intervals, effect sizes, degrees of freedom and *P* value noted<br>*Give P values as exact values whenever suitable.*
☒	☐	For Bayesian analysis, information on the choice of priors and Markov chain Monte Carlo settings
☒	☐	For hierarchical and complex designs, identification of the appropriate level for tests and full reporting of outcomes
☒	☐	Estimates of effect sizes (e.g. Cohen's *d*, Pearson's *r*), indicating how they were calculated

Our web collection on statistics for biologists contains articles on many of the points above.

Software and code

Policy information about availability of computer code

Data collection	Images were captured on a Zeiss LSM880 confocal microscope (Germany) or Olympus fV1000 (Japan). Slice electrophysiology were recorded and captured on a Olympus BX61 (Japan). C-Fos were counted by Photoshop(Adobe, Inc., USA).
Data analysis	Statistical analysis was conducted using Graphpad Prism 8 (Graph Pad Software, Inc., USA), and R 4.2.2 (lucent, Inc., NZ).<br>K-means cluster analysis was conducted using SPSS 16.0 (IBM, Inc., USA).<br>Electrophysiology analysis was conducted using Clampfit 10.4 (Sunnyvale, Inc., USA).

For manuscripts utilizing custom algorithms or software that are central to the research but not yet described in published literature, software must be made available to editors and reviewers. We strongly encourage code deposition in a community repository (e.g. GitHub). See the Nature Portfolio guidelines for submitting code & software for further information.

Data

Policy information about availability of data

All manuscripts must include a data availability statement. This statement should provide the following information, where applicable:

- Accession codes, unique identifiers, or web links for publicly available datasets
- A description of any restrictions on data availability
- For clinical datasets or third party data, please ensure that the statement adheres to our policy

The data that support the fundings of this study can be found in the paper and its supplementary information. Source data are provided with this paper.

Research involving human participants, their data, or biological material

Policy information about studies with human participants or human data. See also policy information about sex, gender (identity/presentation), and sexual orientation and race, ethnicity and racism.

Reporting on sex and gender	This study does not involve human and related clinical studies. This clause does not apply to this study.
Reporting on race, ethnicity, or other socially relevant groupings	This study does not involve human and related clinical studies. This clause does not apply to this study.
Population characteristics	This study does not involve human and related clinical studies. This clause does not apply to this study.
Recruitment	This study does not involve human and related clinical studies. This clause does not apply to this study.
Ethics oversight	This study does not involve human and related clinical studies. This clause does not apply to this study.

Note that full information on the approval of the study protocol must also be provided in the manuscript.

Field-specific reporting

Please select the one below that is the best fit for your research. If you are not sure, read the appropriate sections before making your selection.

☒ Life sciences ☐ Behavioural & social sciences ☐ Ecological, evolutionary & environmental sciences

For a reference copy of the document with all sections, see nature.com/documents/nr-reporting-summary-flat.pdf

Life sciences study design

All studies must disclose on these points even when the disclosure is negative.

Sample size	No statistical methods were used to pre-determine sample sizes but our sample sizes are similar to those reported in previous publication(PMID: 17956738, and are deemed appropriate based on the size and statistical significance of the effect and consistency across animals. Sample sizes are reported in Methods and Supplementary Table.5.
Data exclusions	For all experiments, mice with signs of infection/bleeding/unhealthy conditions after the surgeries were excluded for behavioral tests. After experiments, post hoc histological examination showed that viral injection missed the target region in these animals.
Replication	All behavioral,imaging, and chemogenetics experiments were replicated in multiple animals with similar results (see Supplementary Table 5 for exact numbers of animals and/or trails for each experiment,. Representative immunofluorescent images were based on at least three independent biological samples showing similar results.
Randomization	Mice were randomly assigned to experimental/control groups.
Blinding	All the behavioral experiments of this study were blinded to the behavioral observer. The same batch of experimental mice were labeled with ear tags, and all groups of mice were randomly labeled, The first author presented the animals to the observer for behavioral records. The observer only knew the number of ear tags of each mouse, but did not know the group of mice. All experimental data analysis was blinded like c-Fos counting.

Reporting for specific materials, systems and methods

We require information from authors about some types of materials, experimental systems and methods used in many studies. Here, indicate whether each material, system or method listed is relevant to your study. If you are not sure if a list item applies to your research, read the appropriate section before selecting a response.

Materials & experimental systems

n/a	Involved in the study	
☐	☒	Antibodies
☒	☐	Eukaryotic cell lines
☒	☐	Palaeontology and archaeology
☐	☒	Animals and other organisms
☒	☐	Clinical data
☒	☐	Dual use research of concern
☒	☐	Plants

Methods

n/a	Involved in the study	
☒	☐	ChIP-seq
☒	☐	Flow cytometry
☒	☐	MRI-based neuroimaging

Antibodies

Antibodies used	Primary antibodies used: rabbit anti-c-Fos (1:1000, Cell Signaling Technology, 2250); mouse anti-TH (1:1000, Sigama, MAB318).<br>Secondary antibodies used: anti-mouse Alexa 488 (1:500, Thermo Fisher Scientific, A21202): anti-rabbit Alexa 594 (1:500. Thermo Fisher Scientific, A21207).
Validation	All antibodies are validated for species by the manufacturer.<br>rabbit anti-c-Fos (Cell Signaling Technology, 2250) PMID: 36841874, PMID: 36563678 MORE;<br>mouse anti-TH (Sigama, MAB318) PMID: 35768580, PMID: 34174930 MORE;<br>anti-mouse Alexa 488 (Thermo Fisher Scientific,A21202 PMD: 37250864, PMID: 37389178 MORE;<br>anti-rabbit Alexa 594 (Thermo fisher Scientific, A21207)PMID: 37315138, PMID: 36918560 MORE.

Animals and other research organisms

Policy information about studies involving animals; ARRIVE guidelines recommended for reporting animal research, and Sex and Gender in Research

Laboratory animals	All mice used in the experiments were aged 8 weeks at the start of the experiments/surgeries, These lines are described in the Methods section.<br>Mouse: Vglut2-IRES-Cre:Slc17a6tm2lcre)Lowl/J, 2011 JAX stock 016963.<br>Mouse: Vgat-IRES-Cre: Slc32a1tm2(Cre)Lowl/J, 2011 JAX stock 016962.
Wild animals	No wild animals were used in this study.
Reporting on sex	In this study, we demonstrated that female C57BL/6J mice displayed similar susceptible and resilient phenotypes following the 1-hour social contact process with a similar proportion as observed in the male mice (Supplementary Fig. 2). Any experiments involving female mice have been clarified in the manuscript and the supplementary file, including the sample size. Based on the similar behavioral outcomes in male and females, we did not include females in our brain region screening and functional studies. And we agree that the cellular and circuitry mechanisms underlying the inter-individual differences in females are also essential scientific questions need to be addressed in future independent studies.To avoiding misunderstanding, we made related changes in our title and abstract to clarify that the main findings of the current study are obtained from male mice.
Field-collected samples	No field-collected samples were used in this study.
Ethics oversight	All experiments were performed in accordance with protocols approved by the Animal Care Committee of Xuzhou Medical University (202208S103).

Note that full information on the approval of the study protocol must also be provided in the manuscript.

Plants

Seed stocks	Not applicable.
Novel plant genotypes	Not applicable.
Authentication	Not applicable.
